# Towards an Improvement of Anticancer Activity of Benzyl Adenosine Analogs

**DOI:** 10.3390/molecules26237146

**Published:** 2021-11-25

**Authors:** Verdiana Covelli, Manuela Grimaldi, Rosario Randino, Mohammad Firoznezhad, Maria Chiara Proto, Veronica De Simone, Gianluca Matteoli, Patrizia Gazzerro, Maurizio Bifulco, Anna Maria D’Ursi, Manuela Rodriquez

**Affiliations:** 1 Department of Pharmacy, University of Salerno, Via Giovanni Paolo II, 132-84084 Fisciano, Italy; vcovelli@unisa.it (V.C.); magrimaldi@unisa.it (M.G.); rrandino@unisa.it (R.R.); mfiroznezhad@unisa.it (M.F.); maproto@unisa.it (M.C.P.); pgazzerro@unisa.it (P.G.); 2 Department of Chronic Diseases, Metabolism and Ageing (CHROMETA)-Translational Research Center for Gastrointestinal Disorders (TARGID), KU Leuven, Herestraat, 49-3000 Leuven, Belgium; veronica.desimone@kuleuven.be (V.D.S.); gianluca.matteoli@kuleuven.be (G.M.); 3 Department of Molecular, Medicine and Medical Biotechnology, University of Naples “Federico II”, Via Pansini, 5-80131 Naples, Italy; maurizio.bifulco@unina.it

**Keywords:** N6-benzyladenosine derivatives, colorectal cancer, FPPS

## Abstract

N6-Isopentenyladenosine (**i6A**) is a naturally occurring modified nucleoside displaying in vitro and in vivo antiproliferative and pro-apoptotic properties. In our previous studies, including an in silico inverse virtual screening, NMR experiments and in vitro enzymatic assays, we demonstrated that **i6A** targeted farnesyl pyrophosphate synthase (FPPS), a key enzyme involved in the mevalonate (MVA) pathway and prenylation of downstream proteins, which are aberrant in several cancers. Following our interest in the anticancer effects of FPPS inhibition, we developed a panel of **i6A** derivatives bearing bulky aromatic moieties in the N6 position of adenosine. With the aim of clarifying molecular action of N6-benzyladenosine analogs on the FPPS enzyme inhibition and cellular toxicity and proliferation, herein we report the evaluation of the N6-benzyladenosine derivatives’ (compounds **2a–m**) effects on cell viability and proliferation on HCT116, DLD-1 (human) and MC38 (murine) colorectal cancer cells (CRC). We found that compounds **2**, **2a** and **2c** showed a persistent antiproliferative effect on human CRC lines and compound **2f** exerted a significant effect in impairing the prenylation of RAS and Rap-1A proteins, confirming that the antitumor activity of **2f** was related to the ability to inhibit FPPS activity.

## 1. Introduction

**Figure 1:**
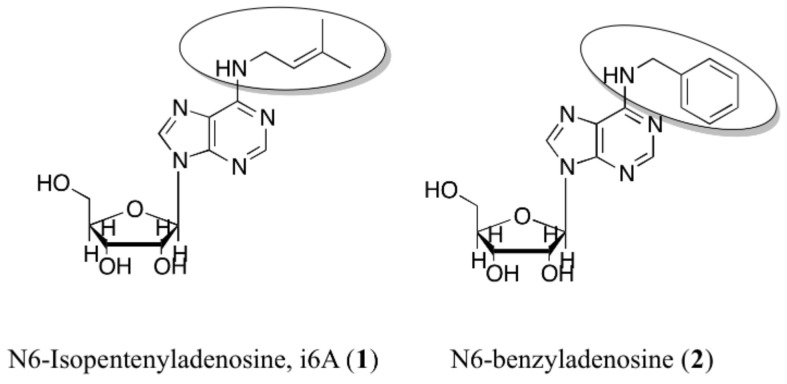
Chemical structures of **i6A** (**1**) and N6-benzyladenosine (**2**).

N6-Isopentenyladenosine (**i6A**) is a modified nucleoside formed by adenosine, harboring an isopentenyl chain derived from dimethylallyl pyrophosphate in the N6 position (Figure 1). It belongs to the cytokinin family, initially discovered as inducers of plant cell division in culture [1]. The cytokinin phytohormones are now recognized as involved in a plethora of regulating plants processes [2,3], such as seed germination, root growth and leaf senescence [4,5,6]. In humans, many biological actions, both in vitro and in vivo, including antitumor effects, can be attributed to **i6A** [7,8,9,10,11,12,13]. For example, we demonstrated that **i6A** exerts antiproliferative effects in thyroid K-RAS (KiMol) transformed cells and untransformed FRTL-5 wild-type cells; in vivo, it shows antiproliferative action by inhibiting the growth of murine xenograft (where cancer KiMol cells were implanted subcutaneously) [14]. N6-benzyladenosine (**2**, Figure 1) and its substituted-benzyl derivatives have been studied as anticancer agents in different cell lines also as molecules capable of complexing transition metals [15,16,17]. N6-benzyladenine, the cytokinin free base form, has been shown to induce cell differentiation -inducing effects at concentrations between 25–100 µmol/L in acute myeloid leukemia cell lines. The growth inhibition and differentiation of cells induced by N6-benzyladenine may require its conversion into nucleotide [18], whereas riboside forms cause apoptosis of leukemia cell lines at much lower µmol/L concentrations [19,20]. It was shown that the N6-cytokinins induced apoptosis after their intracellular phosphorylation and activation of caspases [21,22,23,24]. Moreover, the cytokinins interconversion into their various structural derivatives was tracked in mammalian cells (HeLa), and considering the cytotoxic activity of the riboside **2**, it was expected to induce cytotoxicity at 1 µmol/L concentration. In these cell line however, this effect was not observed and the nucleotide derivative of **2** was barely detected. More importantly, a conversion of **2** into the N6-benzyladenine was observed, hypothesizing that the HeLa cells can circumvent the cytotoxicity of **2** through its modification into a nitrogenous base instead of the nucleotide form [25].

The FPPS enzyme catalyzes the synthesis of several essential metabolites and is critical in protein prenylation and cell membrane synthesis [26,27,28]. In searching for the molecular mechanism explaining the cytotoxic and antiproliferative activity of **i6A**, we found that it modulated FPPS expression and activity in several experiments. In particular, on human natural killer (NK) cells, **i6A** directly stimulated the proliferation, the chemokine secretion, and the cytotoxic activity vs. conventional cancer target cells by the induction of the expression and the activity of FPPS [29,30]. The mechanisms relating the antitumor activity of **i6A** to FPPS are not fully understood; however, several pieces of evidence have shown an **i6A**-FPPS structural interaction. Specifically, the interaction of **i6A** with FPPS was first predicted by us in inverse virtual screening [31] and then proved by NMR experiments [32,33,34,35]. Based on these data, we designed new i6A analogs endowed with improved biological activity. Among these, compound **2** (Figure 1) was confirmed as the most promising **i6A** derivative, characterized by increased cytostatic and antiproliferative activities compared to **i6A**. The N6-benzyladenosine (**2**) selectively targeted glioma cells -with no cytotoxicity on healthy brain cells- inducing intrinsic pathways of apoptosis and inhibition of proliferation. Moreover, it counteracted the oncogenic signaling mediated by the epidermal growth factor receptor (EGFR) [32].

**Figure 2:**
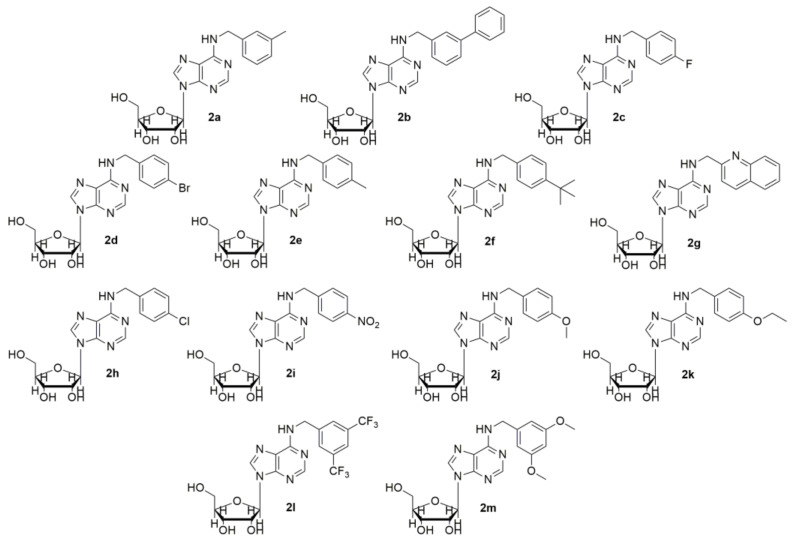
Chemical structures of N6-benzyladenosine derivatives (**2a–m**).

Our molecular docking calculations showed that the isopentenyl moiety and the benzyl ring laid in the allylic sub-pocket of FPPS could contain additional bulky substituents. Intending to probe the FPPS binding site, we designed, synthesized and screened a panel of N6-benzyladenosine derivatives bearing different chemical moieties on the N6-benzyl ring of **2** (**2a–m**, Figure 2) [33].

NMR data and the 3D superposition of the best binding poses derived from the docking calculations indicated that all the designed molecules had a similar orientation and the *para*-substituted benzyl ring (mostly *p*-tertbutyl benzyl, **2f**) engaged in cation-π interactions with F98, F99, Y204, and L100 of the hydrophobic sub-pocket. Indeed, cytostatic activity in the micromolar range of 2a–m was observed on glioma cells, whereas 2 and 2f showed a millimolar FPPS inhibition, indicating that the structural requirements may affect the FPPS enzyme inhibition differently from cellular toxicity and proliferation [12,33,36,37,38].

In the attempt to elucidate the putative molecular mechanism subtending these apparently odd activities of N6-benzyladenosine derivatives, in the present work, we investigate their biomolecular behavior on HCT116, DLD-1 and MC38 colorectal cancer (CRC) cells.

## 2. Results and Discussion

**Figure 3:**
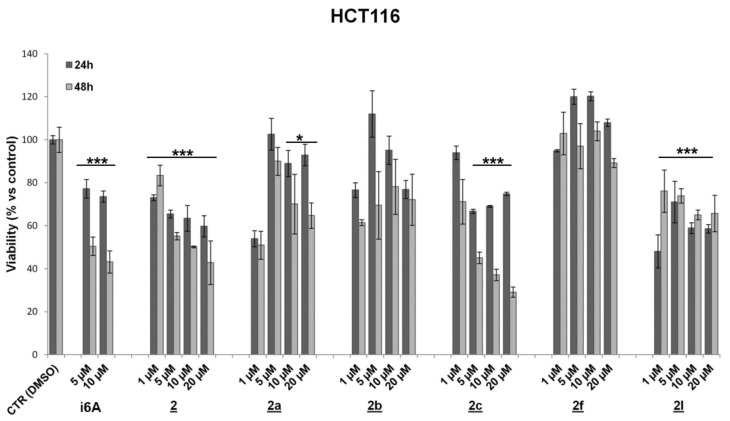
Effect of **2** and its analogues on HCT116 cell viability. Values are means ± SD from three individual experiments (* *p* < 0.05; *** *p* < 0.001; unpaired Student’s *t*-test).

**Figure 4:**
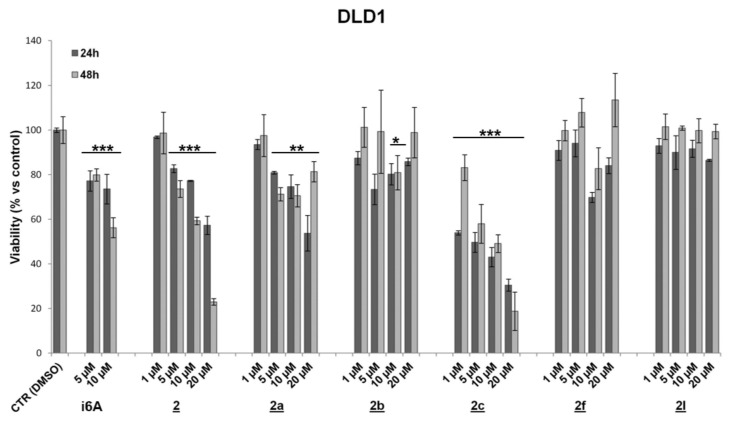
Effect of **2** and its analogues on DLD-1 cell viability. Values are means ± SD from three individual experiments (* *p* < 0.05; ** *p* < 0.01; *** *p* < 0.001; unpaired Student’s *t*-test).

To evaluate drug effects on cell viability and proliferation, HCT116, DLD-1 (human colorectal adenocarcinoma), and MC38 (murine colon adenocarcinoma) cell lines characterized by different genetic profiles and sensitivity to chemotherapies were treated for 24 and 48 h with N6-benzyladenosine (**2**) and its analogs (concentrations ranging from 1 to 20 μM). Assessment of cell viability and proliferation was performed by the MTT assay and IncuCyte live-cell imaging and analysis system. Results showed that the lead compound **2** and the analogs **2a** and **2c** exerted a significant and persistent cytotoxic effect, starting from 24 h and more evident at 48 h, on both human colon cancer cell lines (Figure 3 and Figure 4 and Table 1). On the other hand, these analogs showed no significant effect on murine colon cell line MC38 (Figure S1 in Supplementary Materials).

For other analogs, although some of them (**2g**, **2l**) achieved statistical significance, at least at 10 μM after 48 h treatment, no precise dose or time-dependent cytotoxic effect can be evidenced in all CRC cell lines. In particular, the analogue **2g** showed a significant inhibitory effect of viability in DLD-1 cells and a detectable antiproliferative effect in MC38 at a dose of 10 μM (Figure S2 in Supplementary Materials); the compound **2l** was found to be effective in HCT116 but not both in DLD-1 and MC38, while the analogs **2d**, **2f** and **2g** exerted a slight proliferative effect in HCT116 (Figure 3 and Table 1) and in MC38 for **2f** after 48 h treatment (Figure S2 in Supplementary Materials).

**Figure 5:**
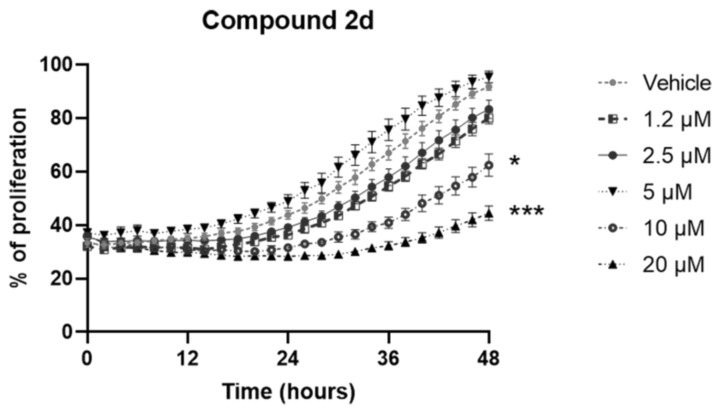
Antiproliferative effect of the analog compound **2d** on MC38 cell line treated for 48 h (1.2–20 μM). Data are expressed as mean values ± SEM. Data sets were compared with two-way analysis of variance (ANOVA) test followed by Dunnett’s correction (* *p* < 0.05, *** *p* < 0.001).

Quite the opposite, the analog **2d** showed a good antiproliferative effect on murine CRC cells at a dose of 10 μM and 20 μM regardless, with no dose-dependent effect. Indeed, at both 24 and 48 h post-treatment with the highest concentrations of **2d** compound, we observed a significant reduction of cell proliferation (Figure 5).

**Figure 6:**
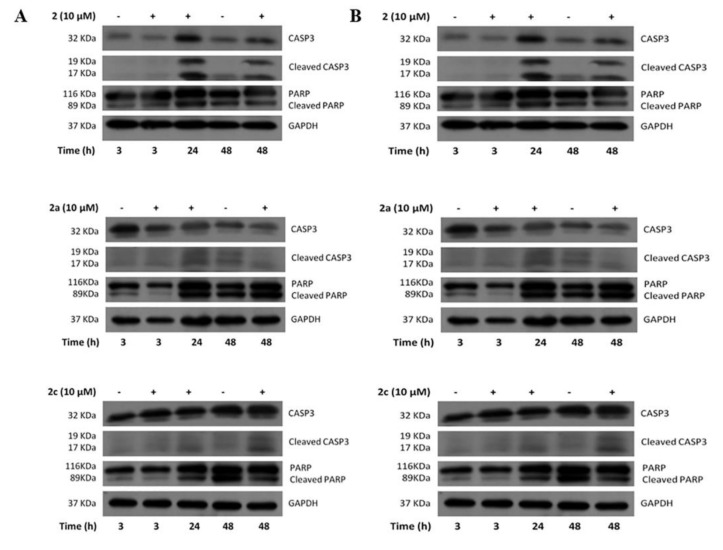
Western blots analysis of caspase-3 and PARP expression performed in DLD-1 cells (**A**) and HCT116 (**B**) treated with compounds **2**, **2a** and **2c**. The apoptotic process was activated by treatment with these three compounds.

To elucidate the mechanisms responsible for the observed cytotoxicity, we analyzed the apoptotic pathway induction through Western blots performed in human CRC cells treated with selected compounds. For this purpose, we focused further analyses on the most promising compounds, such as (i) the lead compound (**2**) and the analogues **2a** and **2c**, (ii) the analogues **2b** and **2l**, able to induce cytotoxicity in DLD-1 or HCT116, respectively, and (iii) the compound **2f** that seemed to be the most promising from NMR and docking analysis, but substantially ineffective in the control of cell viability and cell proliferation. Apoptotic cell death involves activating of the caspase family, and is accomplished through the cleavage of some proteins essential for normal cell maintenance and survival, such as caspase-3 and PARP cleaved forms [16]. According to the previous assay, the lead compound **2**, **2a** and **2c** induced apoptosis highlighted by the increase of caspase-3 and PARP cleaved forms revealed in DLD-1 and HCT116 treated for 24 h and 48 h (Figure 6A,B panels respectively). Conversely, the apoptotic process was not triggered by treatment with **2b**, **2f** or **2l** (Figures S3 and S4 in Supplementary Materials).

**Figure 7:**
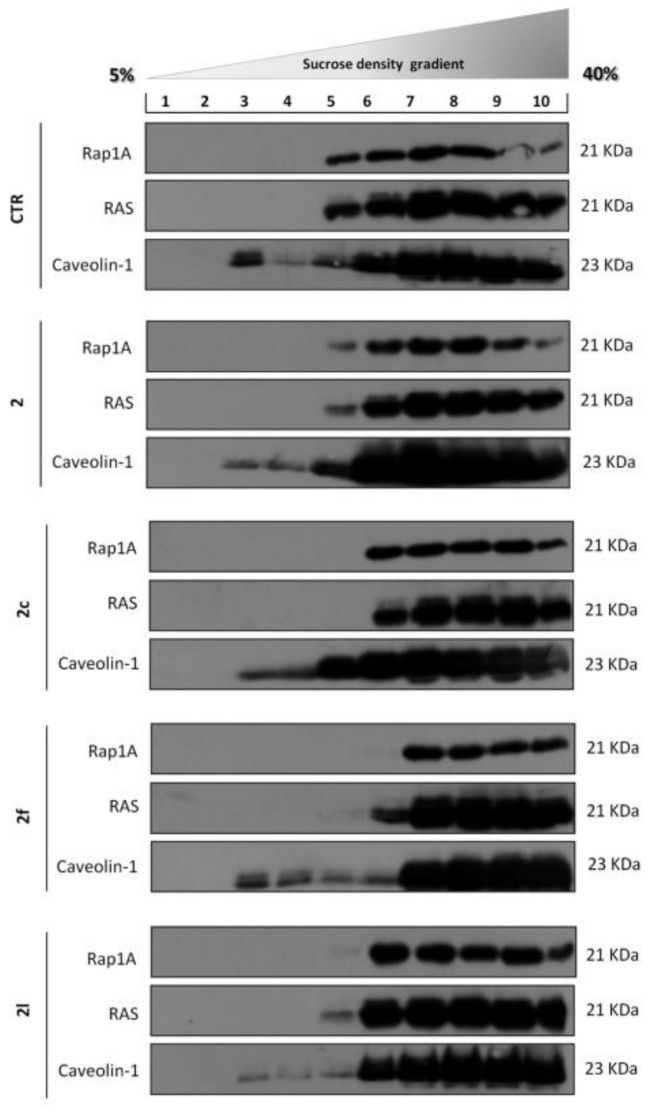
Western blot analysis of sucrose density gradient fractions of HCT116 cells treated with compound **2** and **2a–m** (10 μM for 24 h). **2f** induced an accumulation of both RAS and Rap-1A proteins into the top-heavy fractions of the gradient (6–10).

To prove that the antitumor activity of **i6A** analogs was related to the ability to impair FPPS activity, we measured their effect on protein prenylation in HCT116 cells. Protein prenylation is necessary for membrane localization and the proper function of otherwise cytosolic proteins; therefore, an effect on protein prenylation may have significant consequences on the survival of the cells. However, reliable measurement of protein prenylation in cell lines or tumor specimens is significantly interfered by the multiple post-translational lipid modifications, such as farnesylation and geranylgeranylation, which are specific to each protein, and rapidly occur throughout the cell cycle and in response to selected treatments [39]. Moreover, the lack of commercial antibodies to discriminate prenylated proteins made it impossible to evaluate post-translational modifications by simple biological tests. Thus, protein prenylation was measured by observing through sucrose density centrifugation the localization of Rat Sarcoma Viral Oncogene Homolog (RAS) and RAS-Related Protein Rap-1A (Rap-1A), generally prenylated proteins with membrane localization. If the prenylation is impaired, they are found as soluble proteins in the cytosolic fraction. Quantification of the unprenylated fraction of RAS and Rap-1A through Western blot analysis (Figure 7) relates to the inhibition of protein prenylation. Sucrose density centrifugation of HCT116 cells treated with compound **2** and **2a–m** (10 μM for 24 h), (Figure 7), indicated the ability of **2f** to interfere with protein prenylation and therefore to induce an accumulation of both RAS and Rap1A proteins into the top-heavy fractions of the gradient (6–10), enriched in soluble proteins.

Sucrose density treatment performed on CRC cells confirmed the ability of compound **2f** to inhibit RAS, and Rap-1A prenylation, which was evident in the detachment of the two proteins from the membrane compartments. A less important action was observable for **2c**, which induced the disappearance of the proteins located in fraction 5 of the gradient (Figure 7). In brief, the results confirmed a potent inhibitory effect of the lead compound and some synthetic analogues in CRC cell lines. However, the observed cytotoxic effects do not seem exclusively ascribable to direct inhibition of FPPS, at least in human CRC.

## 3. Materials and Methods

### 3.1. Reagents and Antibodies

N6-Isopentenyladenosine (**i6A**), 3-(N-Morpholino)propanesulfonic acid (MOPS) and NaCl-Sucrose were purchased from Sigma-Aldrich, Inc, St. Louis, MO, USA. The antibodies used: anti-Rap1A, anti-RAS, anti-caspase 3 (total and cleaved forms), anti- PARP (total and cleaved forms) were from Abcam, Cambridge, UK; anti-Caveolin-1 was from Santa Cruz Biotechnology, Dallas, TX, USA; anti-GAPDH and horseradish peroxidase-conjugated secondary antibodies were purchased from Cell Signaling Technologies, Danvers, MA, USA.

### 3.2. Cell Cultures, Treatments and Cell Viability Assay

Human colorectal cancer cell lines HCT116 and DLD-1 were obtained from the Interlab Cell Line Collection (IST, Genoa, Italy) and grown in McCoy’s 5A and RPMI-1640 medium respectively, at 37 °C in a 5% CO_2_ atmosphere. To evaluate cell viability, the colorimetric MTT (3-(4,5 di-methylthiazol-2-yl)-2,5-diphenyltetrazolium bromide) assay was used as previously described [40]. Briefly, HCT116 and DLD-1 cells were seeded at a density of 1 × 10^5^ cells/well and exposed to increasing concentrations of compounds for 24 h or 48 h. MTT stock solution (5 mg/mL in PBS, Sigma) was added to each well and incubated for 4 h at 37 °C in humidified CO_2_. The reduction of tetrazolium to colored formazan only occurred in metabolically active cells and was monitored by spectrophotometer at an optical density of 595 nm after solubilization of the formazan crystals with acidic isopropanol (0.1 N HCl in absolute isopropanol). Each data point represents the average of three separate experiments in triplicate.

### 3.3. Cell Cultures, Treatments and Proliferation Assay

Mouse colon adenocarcinoma cell line MC38 was obtained from American Type Culture Collection (ATCC Catalog #PTA-2920, Manassas, VA, USA) and grown in Dulbecco’s modified MEM (DMEM; Gibco^TM^) supplemented with 10% fetal bovine serum (FBS; Gibco), 2 mM penicillin-streptomycin, 2 mM L-glutamine (Lonza^TM^ BioWhittaker^TM^), 2 mM sodium pyruvate (Gibco^TM^) and 1 mM non-essential amino acids (Gibco^®^ MEM Non-Essential Amino Acids, 100X), at 37 °C in a 5% CO_2_ atmosphere. The proliferation assay was performed according to the IncuCyte^®^ Label-Free Cell Proliferation Assay methodology. Briefly, 5 × 10^3^ cells/well were seeded into 96-well flat bottom plate and incubated for 24 h at 37 °C in 5% humidified CO_2_. By day 1, when the cell confluence was approximately 30–40%, the cells were triggered for 48 h with the vehicle (DMSO) (Gibco^TM^) and different concentrations of N6-benzyladenosine derivatives (**2a–m**) (concentrations range 1,2–20 μM). The 96-well flat bottom plates were placed into the IncuCyte^®^ ZOOM System (Sartorius, Göttingen, Germany) and cell growth was assessed for 48 h, with scan interval every 2 h.

### 3.4. Sucrose Density Gradient

To perform cells fractionation by sucrose density gradient, HCT116 cells were seeded at a concentration of 3.5 × 10^6^ in 100 mm dishes and, when they reached 60–70% confluency, they were treated with 10 µM of the compounds for 24 h. Cells were harvested by Trypsin-EDTA, suspended in Mc Coy’s 5A supplemented with 10% FBS and incubated at 37 °C with 5% CO_2_ for 1 h. About 7.5 × 10^6^ cells were washed three times with ice-cold PBS. Lysis buffer, containing 1% Triton X-100 (AppliChem GmbH, Darmstadt, Germany) and protease/phosphatase inhibitors in MOPS-buffered saline (25 mM 3-(N-Morpholino) propanesulfonic acid, 0.15 M NaCl, pH 6.5), was added to the cell pellet and left 30 min on ice. The lysate was adjusted to 40% sucrose (Sigma-Aldrich, Inc.) by mixing with equal volume of 80% sucrose prepared in MOPS-buffered saline and placed at the bottom of a Beckman ultracentrifuge tube. A discontinuous density gradient was prepared above by layering progressively 30% sucrose solution upon the first one and 5% sucrose solution at the top. Tubes were centrifuged at 32,500 rpm for 20–24 h at 4 °C in an SW-50.1 rotor (Beckman Instruments, Palo Alto, CA, USA). Ten fractions were collected from the top to the bottom of the tube and analyzed by Western blot to determine proteins distribution.

### 3.5. Western Blot Analysis

After treatment, HCT116 and DLD1 cells were washed with PBS, detached using trypsin and collected in ice-cold RIPA Buffer (50 mM Tris-HCl, 150 mM NaCl, 0.5% Triton X-100, 0.5% deoxycholic acid, 10 mg/mL leupeptin, 2 mM phenylmethylsulfonyl fluoride and 10 mg/mL aprotinin). Equal amount of whole cell extracts (15–30 μg of proteins), or equal volume of each sucrose density fraction was loaded on a 10% SDS-PAGE gel and separated by electrophoresis as described previously [19]. Proteins were transferred to nitrocellulose membranes that were blocked with 5% milk (Bio-Rad Laboratories, Inc., Hercules, CA 94547 USA) and probed with specific antibodies, anti-Caveolin-1 as marker of lipid rafts, or anti-GAPDH used as loading controls. Membranes were then incubated with horseradish peroxidase-conjugated secondary antibodies and proteins detected by a chemiluminescence system (Amersham™ ECL™, Sigma-Aldrich S.r.l.).

### 3.6. Chemistry

Lead compound **2** and the analogs **2a–m** were prepared and purified as described previously [15,33,41,42,43,44,45,46]. NMR spectra were recorded at room temperature on Bruker Avance 600 MHz spectrometer. Chemical shifts (δ) are reported in ppm relative to the residual solvent peak (MeOD) and the multiplicity of each signal is designated by the following abbreviations: s, singlet; d, doublet; t, triplet; q, quartet; m, multiplet; br, broad; app, apparent. Coupling constants (J) are quoted in Hz. High resolution mass spectra (HRMS), recorded on a high-resolution mass spectrometer equipped with electrospray (ESI) and nanospray sources, a quadrupole-time of flighthy brid analyser coupled with capillary UPLC system (Q-TOF Premier/nano Aquity, Waters) in positive mode, and protonated molecular ions [M + H]^+^, were used for empirical formula confirmation. Liquid chromatography was performed on a Waters system (Milford, MA, USA) consisting of a Waters 486 tunable absorbance detector and a Varian 9012 pump. Samples were prepared by dissolving compounds **2a–m** in methanol (0.5 mg/mL) at 0.2 mL/min. The injection volume was 10 μL. Compounds **2a–m** were analyzed on a Symmetry^®^ C18 column (4.6 × 250 mm, 5 μm) under gradient elution at a flow rate of 0.9 mL/min. The mobile phases consisted of 2% (*v*/*v*) acetic acid in water (solvent A) and 2% (*v*/*v*) acetic acid in acetonitrile (solvent B). The following gradient was used: 0–20 min 10–90% B, 20–24 min 90–10% B, and return to the initial conditions over 3 min. UV detection was obtained at λ = 254 nm. Purity of all compounds (≥99%) was verified by HPLC, NMR and mass spectrometry measurements. NMR, HRMAS and HPLC details are shown in Supplementary Materials for each compound, reported using the IUPAC nomenclature (Figures S5–S18). Structures of these compounds are presented in Figure 1 and Figure 2.

### 3.7. Statistical Analysis

Significance between two groups was determined by unpaired two-tailed Student’s *t*-test, while two-way analysis of variance (two-way ANOVA) followed by Dunnett’s Multiple comparison test was performed to compare multiple mean groups. Graph Pad Prism 9.2.0 software (GraphPad Software, Inc., San Diego, CA 92108, USA) [47] was used to generate graphs and perform statistical analysis.

## 4. Conclusions

Taken together, our results showed that the lead compound **2** and the analogs **2a** and **2c** exerted a significant and persistent antiproliferative effect on the HCT116, DLD-1 human colorectal adenocarcinoma cell lines, while there was no significant effect on murine colon cell line MC38. By contrast, the analog **2d** showed a good antiproliferative effect on murine CRC cells at a dose of 10 μM and 20 μM. The time and concentration-dependent effect of some analogs, such as **2g** and **2l**, on the cell viability was not observed in all CRC cell lines, although statistical significance was achieved, at least at 10 μM after 48 h treatment. To investigate the molecular mechanism related to the observed cytotoxicity, we analyzed the induction of apoptotic pathways through Western blots performed in human CRC cells. The increased expression of caspase-3 and PARP cleaved forms confirmed that compound **2** and the analogs **2a** and **2c** induced the apoptotic pathway. Finally, to investigate the possibility that the antitumor activity of **2** and **2a–m** compounds would be related to the modulation of the proteins prenylation, RAS and Rap-1A prenylation was measured in HCT116 cell line. Sucrose density centrifugation coupled with Western blot analyses proved that compound **2f** had a significant effect in impairing the prenylation of RAS and Rap-1A proteins, proving that the antitumor activity of **2f** was related to the ability to inhibit FPPS activity. These new results confirmed our previously reported data, highlighting the significant capabilities of benzyl adenosine analogs to exert anticancer activity through FPPS enzyme inhibition. Nevertheless, our apparently contradictory results, with respect to cell viabilities and cytotoxic activity, might be caused by several phenomena corroborating the hypothesis that cytokinins in situ interconversion into their various structural derivatives may occur in a time-dependent manner, mimicking the interconversion hormetic effect. Cellular metabolism or a temporary cell cycle arrest due to the ability of cancer cells to evade the cell-cycle exit somehow might explain our findings. Additionally, unspecific inhibition of regulatory or feedback loops in the mevalonate pathway might contribute to sustaining this process. However, whether and how our compounds exhibit a hormetic effect through the mevalonate pathway need to be further investigated.

## Figures and Tables

**Table 1:** Distribution of cell viability and proliferation percentages at different time points and at 10 μM concentration in human (HCT116, DLD-1) and murine (MC38) colorectal adenocarcinoma cell lines.

Compound (10 μM)	Cell Viability (%) ± SD				Cell Proliferation (%) ± SD	
	DLD-1		HCT116		MC38	
	24 h	48 h	24 h	48 h	24 h	48 h
**1**	73.6 ± 6.6	56.2 ± 4.5 ***	73.0 ± 2.6	43.2 ± 5.1 ***	88.5 ± 4.4	99.8 ± 0.2
**2**	77.3 ± 0.3	59.3 ± 1.7 ***	63.5 ± 5.9	50.2 ± 0.5 ***	73.7 ± 5.9	98.0 ± 0.7 **
**2a**	74.6 ± 5.3	70.6 ± 5.0 **	88.9 ± 6.1	70.1 ± 13.9 *	93.6 ± 3.1 *	99.9 ± 0.03
**2b**	80.2 ± 4.8	80.9 ± 7.7 *	95.1 ± 6.6	78.1 ± 12.8	53.3 ± 5.0 *	98.5 ± 1.2
**2c**	43.1 ± 4.4	49.1 ± 4.0 ***	69.0 ± 0.5	37.1 ± 2.7 ***	69.1 ± 6.0	99.3 ± 0.5
**2d**	79.9 ± 1.8	72.3 ± 14.1 *	127.8 ± 0.8	96.9 ± 8.7	31.5 ± 1.7 **	62.3 ± 7.3 *
**2e**	80.7 ± 2.5	113.6 ± 3.5 *	70.0 ± 4.7	95.1 ± 1.5	67.5 ± 3.6	99.9 ± 0.1
**2f**	69.8 ± 2.3	82.7 ± 9.4	120.2 ± 2.1	103.9 ± 4.3	67.9 ± 6.9	98.5 ± 0.8
**2g**	53.9 ± 1.8	89.3 ± 3.5	108.7 ± 2.5	97.8 ± 5.8	64.6 ± 6.1 *	99.1 ± 1.4
**2h**	90.9 ± 5.5	90.4 ± 2.9	65.9 ± 26.6	92.9 ± 1.1	90.0 ± 5.9	99.9 ± 0.03
**2i**	75.1 ± 1.2	72.0 ± 12.9 *	52.0 ± 13.3	72.9 ± 18.2	84.0 ± 5.4 *	99.9 ± 0.1
**2j**	84.3 ± 4.4	101.2 ± 1.3	60.4 ± 19.1	80.0 ± 3.1 **	43.1 ± 3.5 *	75.7 ± 4.6 *
**2k**	69.9 ± 4.5	80.9 ± 13.3	91.8 ± 7.7	98.1 ± 3.5	87.9 ± 8.9 *	99.9 ± 0.01 *
**2l**	91.6 ± 3.8	99.8 ± 5.4	58.9 ± 2.5	65.1 ± 2.3 ***	64.6 ± 1.4	99.8 ± 0.01
**2m**	87.9 ± 0.7	73.3 ± 8.1 *	88.0 ± 17.9	92.2 ± 7.4	97.7 ± 0.9	100 ± 0.0004

Results are expressed as percentage (mean ± SD) versus control (* *p* < 0.05; ** *p* < 0.01; *** *p* < 0.001; unpaired Student’s *t*-test).

## Data Availability

The data presented in this study are available online in supplementary material.

## Supplementary Materials

The following are available online, Figure S1. Assessment of cell proliferation was performed by IncuCyte live-cell imaging and analysis system. Results showed that compounds **2a** and **2c** showed no significant effect on murine colon cell line MC38. Data are expressed as mean values ± SEM. Data sets were compared with two-way analysis of variance (ANOVA) test followed by Dunnett’s correction; Figure S2. Assessment of cell proliferation was performed by IncuCyte live-cell imaging and analysis system for **2f** and **2g** compounds. Results showed that compound **2g** displayed detectable antiproliferative effect in MC38 at the dose of 10 μM (* *p* < 0.05) and 20 μM (*** *p* < 0.001) at early time point (24 h). On the contrary, the compound **2f** exerted a slight proliferative effect after 48 h of treatment. Data are expressed as mean values ± SEM. Data sets were compared with two-way analysis of variance (ANOVA) test followed by Dunnett’s correction; Figure S3. Western blots analysis of caspase-3 and PARP expression performed in DLD-1 cell line. The apoptotic process was not triggered by treatment with **2b**, **2f** or **2l**; Figure S4. Western blots analysis of caspase-3 and PARP expression performed in HCT116 cell line. The apoptotic process was not triggered by treatment with **2b**, **2f** or **2l**; Figures S5–S18. 2D HSQC and 2D HMBC NMR experiments in MeOD solvent allowed the ^1^H and ^13^C chemical shift assignment of **2** and **2a-m** compounds proton and carbon signals.

## Sample Availability

Samples of the compounds are available from the authors.

## References

[1] Miller C.O., Skoog F., Von Saltza M.H., Strong F. (1955). Kinetin, a cell division factor from deoxyribonucleic acid1. J. Am. Chem. Soc..

[2] Kersten H. (1984). On the biological significance of modified nucleosides in tRNA. Prog. Nucleic Acid Res. Mol. Biol..

[3] Laten H.M., Zahareas-Doktor S. (1985). Presence and source of free isopentenyladenosine in yeasts. Proc. Natl. Acad. Sci. USA.

[4] Schaller G.E., Bishopp A., Kieber J.J. (2015). The yin-yang of hormones: Cytokinin and auxin interactions in plant development. Plant Cell.

[5] Chen C.-m. (1997). Cytokinin biosynthesis and interconversion. Physiol. Plant..

[6] Mok M.C., Mok D.W.S., Mokeds M.C. (1994). Cytokinins and plant development—An overview. Cytokinin: Chemistry, Activity and Function.

[7] Castiglioni S., Casati S., Ottria R., Ciuffreda P., AM Maier J. (2013). N6-isopentenyladenosine and its analogue N6-benzyladenosine induce cell cycle arrest and apoptosis in bladder carcinoma T24 cells. Anti-Cancer Agents Med. Chem..

[8] Laezza C., Caruso M., Gentile T., Notarnicola M., Malfitano A., Di Matola T., Messa C., Gazzerro P., Bifulco M. (2014). N6-isopentenyladenosine inhibits cell proliferation and induces apoptosis in a human colon cancer cell line DLD1. Int. J. Cancer.

[9] Laezza C., D’Alessandro A., Di Croce L., Picardi P., Ciaglia E., Pisanti S., Malfitano A.M., Comegna M., Faraonio R., Gazzerro P. (2015). p53 regulates the mevalonate pathway in human glioblastoma multiforme. Bioorg. Chem..

[10] Pisanti S., Picardi P., Ciaglia E., Margarucci L., Ronca R., Giacomini A., Malfitano A.M., Casapullo A., Laezza C., Gazzerro P. (2014). Antiangiogenic effects of N6-isopentenyladenosine, an endogenous isoprenoid end product, mediated by AMPK activation. FASEB J..

[11] Ciaglia E., Abate M., Laezza C., Pisanti S., Vitale M., Seneca V., Torelli G., Franceschelli S., Catapano G., Gazzerro P. (2017). Antiglioma effects of N 6-isopentenyladenosine, an endogenous isoprenoid end product, through the downregulation of epidermal growth factor receptor. Int. J. Cancer.

[12] Eun S.Y., Kim H.J., Kang E.S., Kim H.J., Lee J.H., Chang K.C., Kim J.-H., Hong S.-C., Seo H.G. (2010). Farnesyl diphosphate synthase attenuates paclitaxel-induced apoptotic cell death in human glioblastoma U87MG cells. Neurosci. Lett..

[13] Bifulco M., Malfitano A.M., Proto M.C., Santoro A., Caruso M.G., Laezza C. (2008). Biological and pharmacological roles of N6-isopentenyladenosine: An emerging anticancer drug. Anti-Cancer Agents Med. Chem..

[14] Laezza C., Notarnicola M., Caruso M.G., Messa C., Macchia M., Bertini S., Minutolo F., Portella G., Fiorentino L., Stingo S. (2006). N6-isopentenyladenosine arrests tumor cell proliferation by inhibiting farnesyl diphosphate synthase and protein prenylation. FASEB J..

[15] Štarha P., Popa I., Trávníček Z., Vančo J. (2013). N6-Benzyladenosine derivatives as novel N-donor ligands of platinum (II) dichlorido complexes. Molecules.

[16] Dolezal K., Popa I., Zatloukal M., Lenobel R., Hradecká D., Vojtesek B., Uldrijan S., Mlejnek P., Werbrouck S., Strnad M. (2012). Substitution Derivatives of N6-benzyladenosine, Methods of Their Preparation, Their Use for Preparation of Drugs, Cosmetic Preparations and Growth Regulators, Pharmaceutical Preparations, Cosmetic Preparations and Growth Regulators Containing These Compounds. U.S. Patent.

[17] Dolezel P., Koudelkova P., Mlejnek P. (2010). Halogenation of N6-benzyladenosine decreases its cytotoxicity in human leukemia cells. Toxicol. Vitr..

[18] Ishii Y., Sakai S., Honma Y. (2003). Cytokinin-induced differentiation of human myeloid leukemia HL-60 cells is associated with the formation of nucleotides, but not with incorporation into DNA or RNA. Biochim. Et Biophys. Acta -Mol. Cell Res..

[19] Ishii Y., Hori Y., Sakai S., Honma Y. (2002). Control of differentiation and apoptosis of human myeloid leukemia cells by cytokinins and cytokinin nucleosides, plant redifferentiation-inducing hormones. Cell Growth Differ. -Publ. Am. Assoc. Cancer Res..

[20] Mlejnek P. (2001). Caspase inhibition and N6-benzyladenosine-induced apoptosis in HL-60 cells. J. Cell. Biochem..

[21] Voller J., Zatloukal M., Lenobel R., Doležal K., Béreš T., Kryštof V., Spíchal L., Niemann P., Džubák P., Hajdúch M. (2010). Anticancer activity of natural cytokinins: A structure–activity relationship study. Phytochemistry.

[22] Voller J., Béres T., Zatloukal M., Kaminski P.A., Niemann P., Doležal K., Džubák P., Hajdúch M., Strnad M. (2017). The natural cytokinin 2OH3MeOBAR induces cell death by a mechanism that is different from that of the “classical” cytokinin ribosides. Phytochemistry.

[23] Mlejnek P., Doležel P. (2005). Apoptosis induced by N6-substituted derivatives of adenosine is related to intracellular accumulation of corresponding mononucleotides in HL-60 cells. Toxicol. Vitr..

[24] Hertz N.T., Berthet A., Sos M.L., Thorn K.S., Burlingame A.L., Nakamura K., Shokat K.M. (2013). A neo-substrate that amplifies catalytic activity of parkinson’s-disease-related kinase PINK1. Cell.

[25] Aoki M.M., Seegobin M., Kisiala A., Noble A., Brunetti C., Emery R.N. (2019). Phytohormone metabolism in human cells: Cytokinins are taken up and interconverted in HeLa cell culture. FASEB BioAdv..

[26] Sacchettini J.C., Poulter C.D. (1997). Creating isoprenoid diversity. Science.

[27] Szkopińska A., Płochocka D. (2005). Farnesyl diphosphate synthase; regulation of product specificity. Acta Biochim. Pol..

[28] Dhar M.K., Koul A., Kaul S. (2013). Farnesyl pyrophosphate synthase: A key enzyme in isoprenoid biosynthetic pathway and potential molecular target for drug development. New Biotechnol..

[29] Ciaglia E., Pisanti S., Picardi P., Laezza C., Malfitano A.M., D’Alessandro A., Gazzerro P., Vitale M., Carbone E., Bifulco M. (2013). N6-isopentenyladenosine, an endogenous isoprenoid end product, directly affects cytotoxic and regulatory functions of human NK cells through FDPS modulation. J. Leukoc. Biol..

[30] Han S., Li X., Xia Y., Yu Z., Cai N., Malwal S.R., Han X., Oldfield E., Zhang Y. (2019). Farnesyl pyrophosphate synthase as a target for drug development: Discovery of natural-product-derived inhibitors and their activity in pancreatic cancer cells. J. Med. Chem..

[31] Scrima M., Lauro G., Grimaldi M., Di Marino S., Tosco A., Picardi P., Gazzerro P., Riccio R., Novellino E., Bifulco M. (2014). Structural Evidence of N 6-Isopentenyladenosine as a New Ligand of Farnesyl Pyrophosphate Synthase. J. Med. Chem..

[32] Ciaglia E., Grimaldi M., Abate M., Scrima M., Rodriquez M., Laezza C., Ranieri R., Pisanti S., Ciuffreda P., Manera C. (2017). The isoprenoid derivative N6-benzyladenosine CM223 exerts antitumor effects in glioma patient-derived primary cells through the mevalonate pathway. Br. J. Pharmacol..

[33] Grimaldi M., Randino R., Ciaglia E., Scrima M., Buonocore M., Stillitano I., Abate M., Covelli V., Tosco A., Gazzerro P. (2020). NMR for screening and a biochemical assay: Identification of new FPPS inhibitors exerting anticancer activity. Bioorg. Chem..

[34] Mayer M., Meyer B. (1999). Characterization of ligand binding by saturation transfer difference NMR spectroscopy. Angew. Chem. Int. Ed..

[35] Mayer M., Meyer B. (2001). Group epitope mapping by saturation transfer difference NMR to identify segments of a ligand in direct contact with a protein receptor. J. Am. Chem. Soc..

[36] Kim H.Y., Kim D.K., Bae S.-H., Gwak H., Jeon J.H., Kim J.K., Lee B.I., You H.J., Shin D.H., Kim Y.-H. (2018). Farnesyl diphosphate synthase is important for the maintenance of glioblastoma stemness. Exp. Mol. Med..

[37] Ahmad F., Sun Q., Patel D., Stommel J.M. (2019). Cholesterol metabolism: A potential therapeutic target in glioblastoma. Cancers.

[38] Abate M., Laezza C., Pisanti S., Torelli G., Seneca V., Catapano G., Montella F., Ranieri R., Notarnicola M., Gazzerro P. (2017). Deregulated expression and activity of Farnesyl Diphosphate Synthase (FDPS) in Glioblastoma. Sci. Rep..

[39] Berndt N., Hamilton A.D., Sebti S.M. (2011). Targeting protein prenylation for cancer therapy. Nat. Rev. Cancer.

[40] Gazzerro P., Malfitano A.M., Proto M.C., Santoro A., Pisanti S., Caruso M.G., Notarnicola M., Messa C., Laezza C., Misso G. (2010). Synergistic inhibition of human colon cancer cell growth by the cannabinoid CB1 receptor antagonist rimonabant and oxaliplatin. Oncol. Rep..

[41] Kim Y.A., Sharon A., Chu C.K., Rais R.H., Al Safarjalani O.N., Naguib F.N., el Kouni M.H. (2007). Synthesis, biological evaluation and molecular modeling studies of N6-benzyladenosine analogues as potential anti-toxoplasma agents. Biochem. Pharmacol..

[42] Ravn J., Qvortrup K., Rosenbohm C., Koch T. (2007). Design, synthesis, and biological evaluation of LNA nucleosides as adenosine A3 receptor ligands. Bioorg. Med. Chem..

[43] Fujii T., Saito T. (1985). Purines. XXVI. The Dimroth Rearrangement of 9-Substituted 1-Methyladenines: Accelerating Effect of a β-D-Ribofuranosyl Group at the 9-Position. Chem. Pharm. Bull..

[44] Ottria R., Casati S., Baldoli E., Maier J.A., Ciuffreda P. (2010). N6-Alkyladenosines: Synthesis and evaluation of in vitro anticancer activity. Bioorg. Med. Chem..

[45] Randino R., Cini E., D’Ursi A.M., Novellino E., Rodriquez M. (2015). Facile Baeyer–Villiger oxidation of cyclic ketones: Conventional versus microwave-assisted approach. Tetrahedron Lett..

[46] de la Hoz A., Diaz-Ortiz A., Moreno A. (2005). Microwaves in organic synthesis. Thermal and non-thermal microwave effects. Chem. Soc. Rev..

[47] GP Prism (1994). GraphPad Software.

