# Supplementary material for: Towards an Improvement of Anticancer Activity of Benzyl Adenosine Analogs
Source: Molecules. 2021 Nov 25;26(23):7146. doi: 10.3390/molecules26237146 (PMC8658949; doi:10.3390/molecules26237146)
Supplement: Supplementary file 1 [file molecules-26-07146-s001.zip › molecules-1461662-supplementary.pdf]

SUPPORTING INFORMATION

Towards an improvement of anticancer activity of benzyl adenosine analogs

Verdiana Covelli [1], Manuela Grimaldi [1], Rosario Randino [1], Mohammad Firoznezhad [1], Maria Chiara Proto [1], Veronica De Simone [2], Gianluca Matteoli [2], Patrizia Gazzerro [1], Maurizio Bifulco [3], Anna Maria D'Ursi [1,*] and Manuela Rodriquez [1,*]

1 Department of Pharmacy, University of Salerno, Via Giovanni Paolo II, 132 - 84084 Fisciano, Salerno, Italy E-mails: vcovelli@unisa.it (V.C.); magrimaldi@unisa.it (M.G.); rrandino@unisa.it (R.R.); mfiroznezhad@unisa.it (MF); maproto@unisa.it (M.C.P.); pgazzerro@unisa.it (P.G.)
2 Department of Chronic Diseases, Metabolism and Ageing (CHROMETA)-Translational Research Center for Gastrointestinal Disorders (TARGID), KU Leuven, Herestraat ,49 3000 Leuven, Belgium. E-Mails: veronica.desimone@kuleuven.be (V.D.S); gianluca.matteoli@kuleuven.be (G.M.)
3 Department of Molecular Medicine and Medical Biotechnology, University of Naples "Federico II", Via Pansini, 80131, Naples, Italy; E-Mails: maurizio.bifulco@unina.it (M.B.)
*Correspondence dursi@unisa.it (A.M.D.); Tel.: +39 089969748 (Department of Pharmacy, University of Salerno, Via Giovanni Paolo II, 132 - 84084 Fisciano, Salerno, Italy).

Table of context

- **Figure S1.** Assessment of cell proliferation was performed by IncuCyte live-cell imaging and analysis system. Results showed that compounds **2a** and **2c** showed no significant effect on murine colon cell line MC38. Data are expressed as mean values ± SEM. Data sets were compared with two-way analysis of variance (ANOVA) test followed by Dunnett's correction.

- **Figure S2.** Assessment of cell proliferation was performed by IncuCyte live-cell imaging and analysis system for **2f** and **2g** compounds. Results showed that compound **2g** displayed detectable antiproliferative effect in MC38 at the dose of 10 μM (*P < 0.05) and 20 μM (***P < 0.001) at early time point (24 h). On the contrary, the compound **2f** exerted a slight proliferative effect after 48 h of treatment. Data are expressed as mean values ± SEM. Data sets were compared with two-way analysis of variance (ANOVA) test followed by Dunnett's correction.

- **Figure S3.** Western blots analysis of caspase-3 and PARP expression performed in DLD-1 cell line. The apoptotic process was not triggered by treatment with **2b**, **2f** or **2l**.

- **Figure S4.** Western blots analysis of caspase-3 and PARP expression performed in HCT116 cell line. The apoptotic process was not triggered by treatment with **2b**, **2f** or **2l**.

- **Figure S5-S18.** 2D HSQC and 2D HMBC NMR experiments in MeOD solvent allowed the ^{1}H and ^{13}C chemical shift assignment of **2** and **2a-m** compounds proton and carbon signals.

Figure S1: Assessment of cell proliferation was performed by IncuCyte live-cell imaging and analysis system. Results showed that compounds **2a** and **2c** showed no significant effect on murine colon cell line MC38. Data are expressed as mean values ± SEM. Data sets were compared with two-way analysis of variance (ANOVA) test followed by Dunnett's correction.

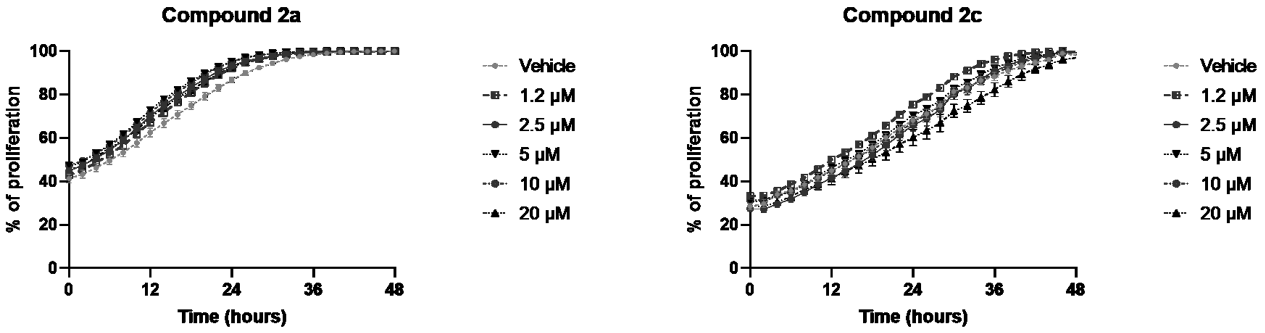

Figure S2: Assessment of cell proliferation was performed by IncuCyte live-cell imaging and analysis system for **2f** and **2g** compounds. Results showed that compound **2g** displayed detectable antiproliferative effect in MC38 at the dose of 10 μM (*P < 0.05) and 20 μM (***P < 0.001) at early time point (24 h). On the contrary, the compound **2f** exerted a slight proliferative effect after 48 h of treatment. Data are expressed as mean values ± SEM. Data sets were compared with two-way analysis of variance (ANOVA) test followed by Dunnett's correction.

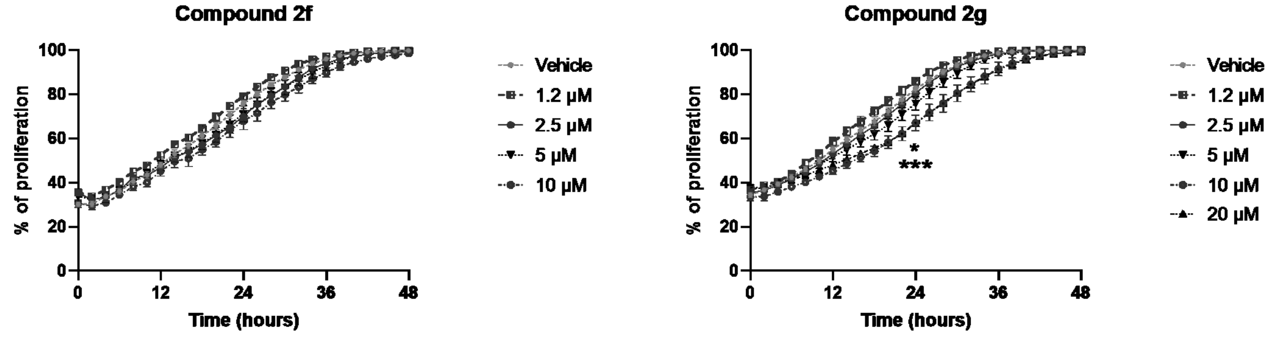

Figure S3: Western blots analysis of caspase-3 and PARP expression performed in DLD-1 cell line. The apoptotic process was not triggered by treatment with **2b**, **2f** or **2l**.

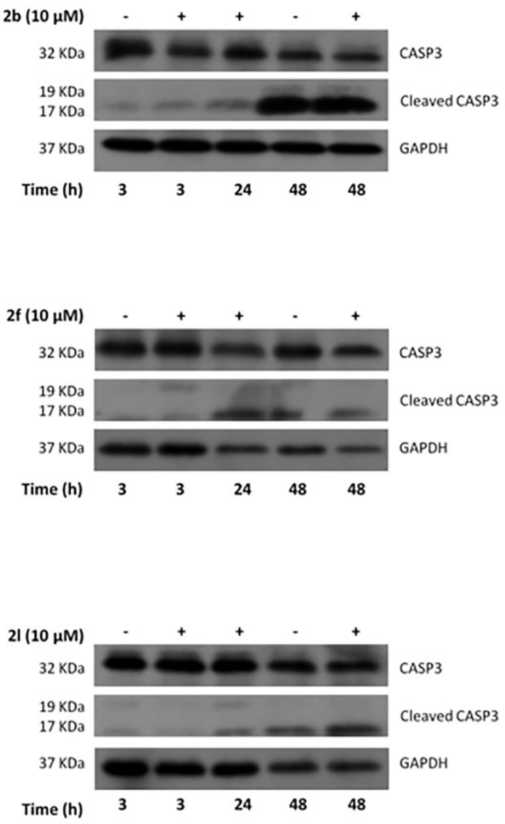

Figure S4: Western blots analysis of caspase-3 and PARP expression performed in HCT116 cell line. The apoptotic process was not triggered by treatment with **2b**, **2f** or **2l**.

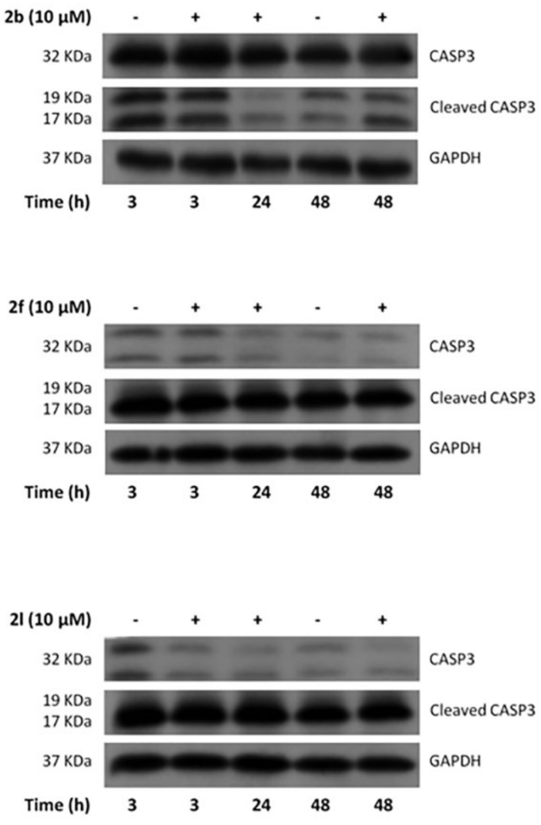

Figure S5: Structure of ((2R, 3R, 4S, 5R)-2-(6-(benzylamino)-9H-purin-9-yl)-5-(hydroxymethyl) tetrahydrofuran-3,4-diol) (**2**). Overlap of 2D-HMBC spectrum in blue, 2D-HSQC in red/fuchsia and 1D ^{1}H spectrum in green.

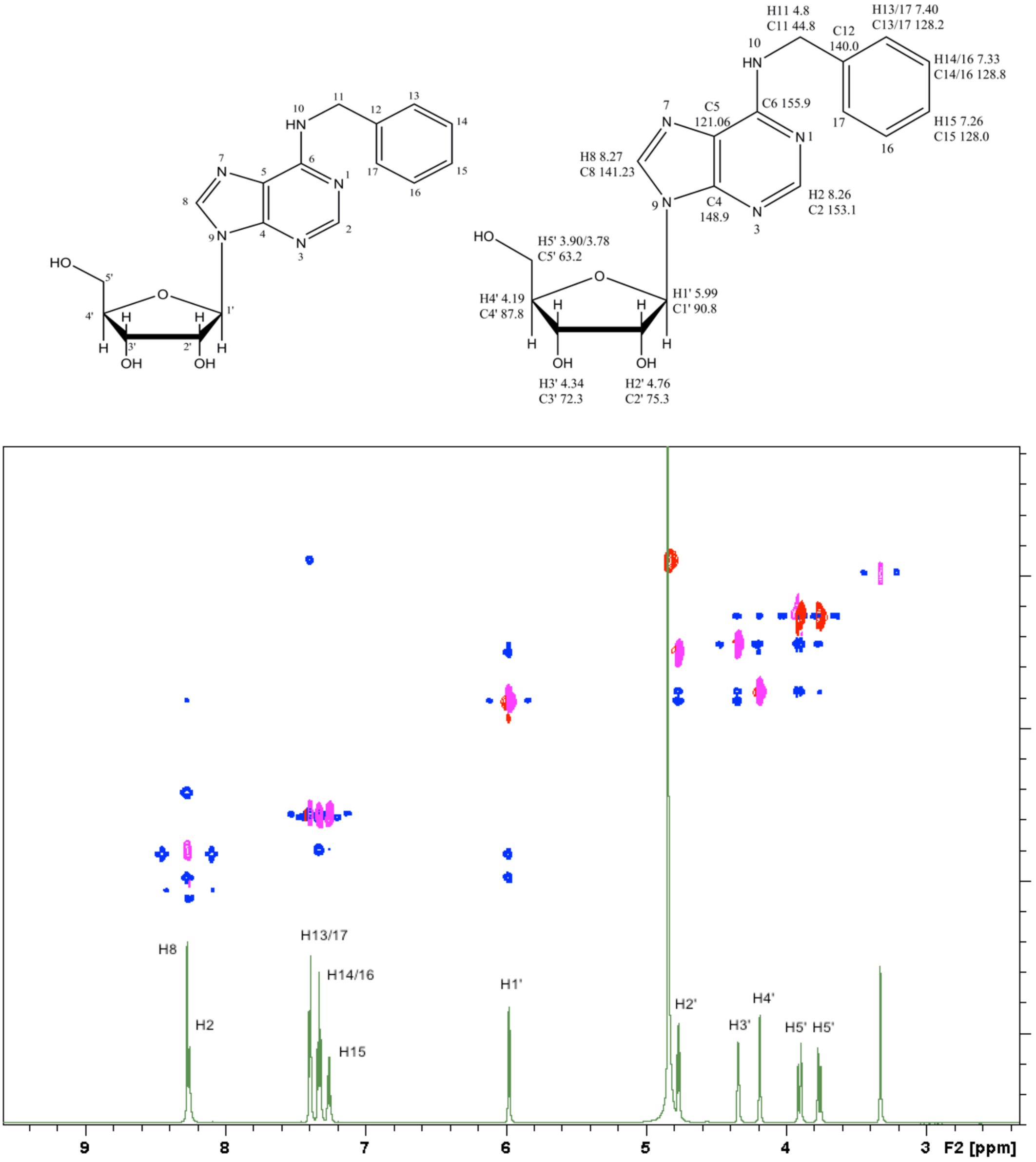

^{1}H NMR (600 MHz, MeOD): δ 8.30 (s, 1H), 8.28 (s, 1H), 7.43 (d, J = 7.5 Hz, 2H), 7.36 (t, J = 7.6 Hz, 2H), 7.29 (t, J = 7.3 Hz, 1H), 6.01 (d, J = 6.4 Hz, 1 H), 4.82–4.77 (m, 1H), 4.37(dd, J = 4.9, 2.5 Hz, 1H), 4.22 (d, J = 2.4 Hz, 1H), 3.93 (dd, J = 12.5, 2.3 Hz, 1H), 3.79 (dd, J =1 2.5, 2.5 Hz, 1H) ppm. ^{13}C NMR (126 MHz, MeOD): δ 155.9, 153.1, 148.9, 141.2, 140.0, 128.8, 128.2, 128.0, 121.0, 90.8, 87.8, 75.3, 72.3, 63.2, 44.8. HRMS (ESI-Q-TOF) m/z $[M+H]^+$ calcd for $C_{17}H_{20}N_5O_4$: 358.1510; found 358.1515. Rt: 8.92 min.

Figure S6: Structure of ((2R,3S,4R,5R)-2-(hydroxymethyl)-5-(6-((3-methylbenzyl)amino)-9H-purin-9-yl)tetrahydrofuran-3,4-diol) (**2a**). Overlap of 2D-HMBC spectrum in blue, 2D-HSQC in red/fuchsia and 1D ^{1}H spectrum in green.

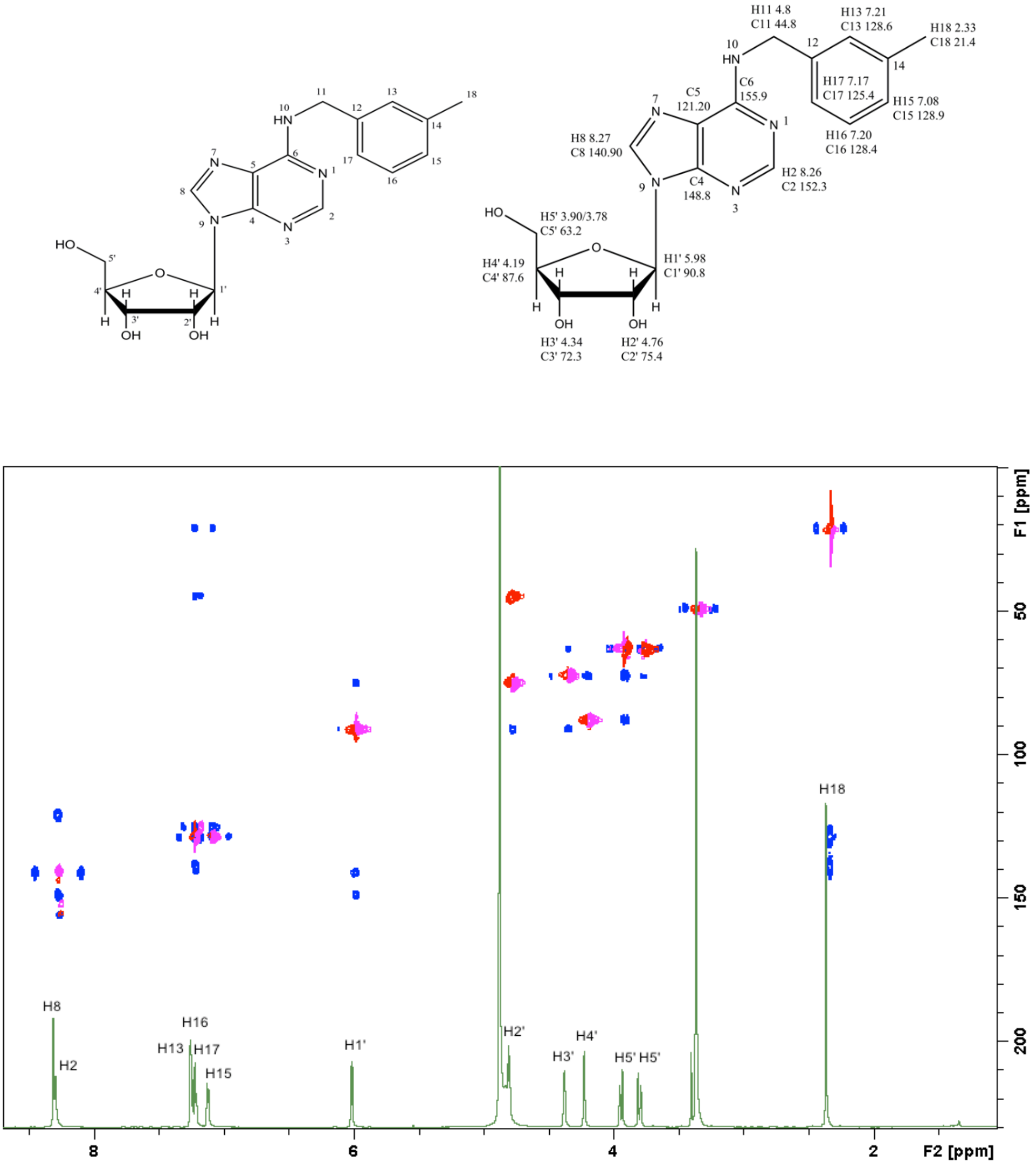

^{1}H NMR (600 MHz, MeOD): δ 8.29 (d, J = 10.4 Hz, 2H), 7.22 (dd, J = 19.2, 9.3 Hz, 3H), 7.11 (d, J = 7.1 Hz, 1H), 6.01 (d, J = 6.3 Hz, 1H), 4.80 (dd, J = 13.0, 7.1 Hz, 3H), 4.39–4.36 (m, 1H), 4.22 (s, 1H), 3.93 (d, J = 12.5 Hz, 1H), 3.79 (d, J =12.5Hz, 1H) ppm. ^{13}C NMR (126 MHz, MeOD): δ 155.9, 152.3, 148.8, 140.9, 128.9, 128.6, 128.4, 121.2, 90.8, 87.6, 75.4, 72.3, 63.2, 44.8, 21.4. HRMS (ESI-Q-TOF) m/z $[M+H]^{+}$ calcd for $C_{18}H_{22}N_5O_4$: 372.1666; found 372.1659. Rt: 9.59 min.

Figure S7: Structure of ((2R,3R,4S,5R)-2-(6-(([1,1′-biphenyl]-3-ylmethyl)amino)-9H-purin-9-yl)-5-(hydroxymethyl)tetrahydrofuran-3,4-diol) (**2b**). Overlap of 2D-HMBC spectrum in blue, 2D-HSQC in red/fuchsia and 1D ^{1}H spectrum in green.

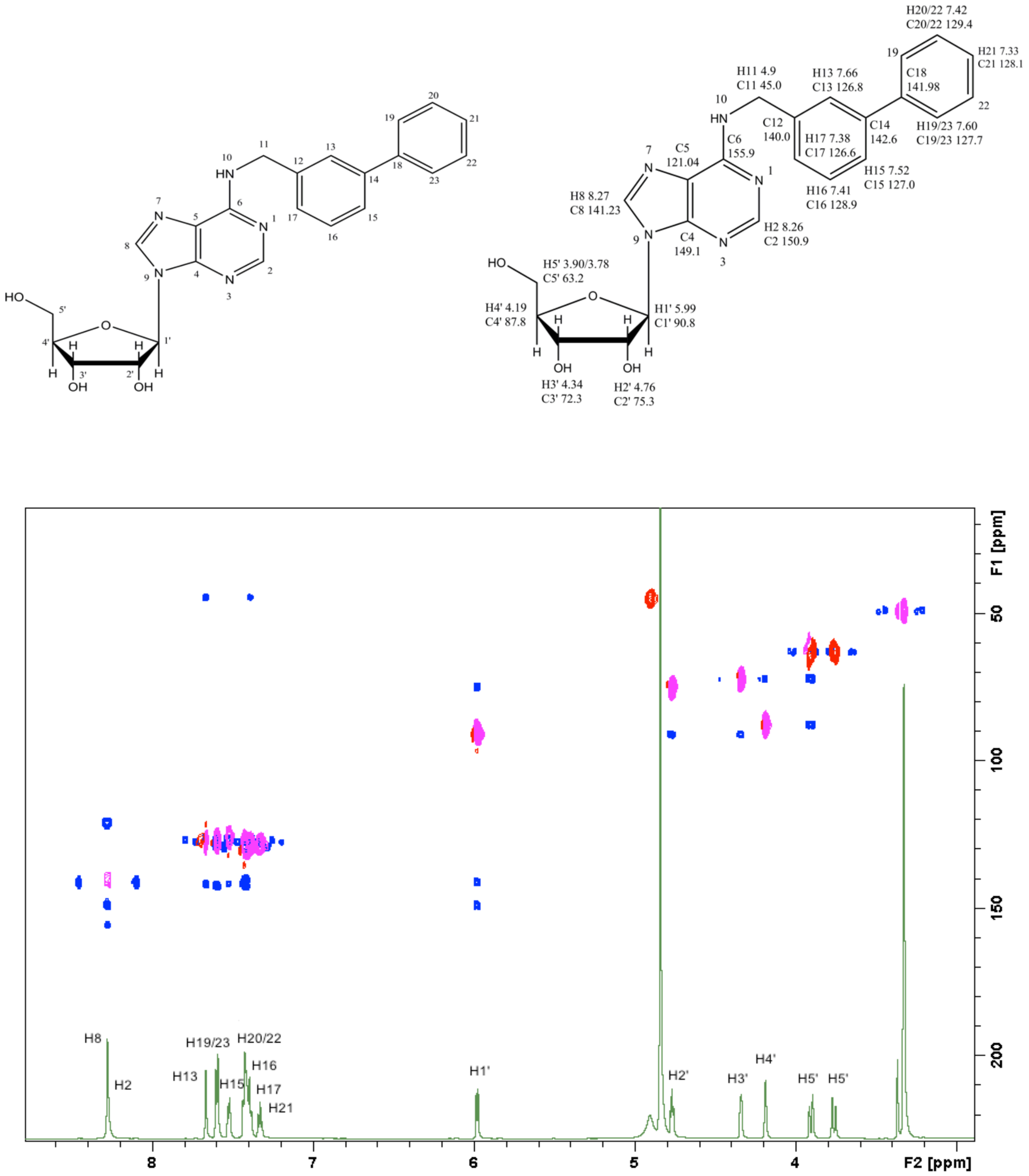

^{1}H NMR (600 MHz, MeOD): δ 8.31 (s, 2H), 7.70 (s, 1H), 7.63 (d, J = 7.5 Hz, 2H), 7.55 (d, J = 7.2 Hz, 1H), 7.44 (dt, J = 18.8, 7.2 Hz, 4H), 7.36 (t, J = 7.2 Hz, 1H), 6.01 (d, J = 6.3 Hz, 1H), 4.80 (t, J = 5.6 Hz, 1H), 4.39–4.35 (m, 1H), 4.22 (s, 1H), 3.93 (d, J = 12.5 Hz, 1H), 3.79 (d, J = 12.5 Hz, 1H) ppm. ^{13}C NMR (126 MHz, MeOD): δ 155.9, 159.9, 149.1, 142.6, 141.9, 141.2, 140.0, 129.4, 128.9, 128.1, 127.7, 127.0, 126.8, 126.6, 121.0, 90.8, 87.8, 75.3, 72.3, 63.2, 45.0. HRMS (ESI-Q-TOF) m/z $[M+H]^+$ calcd for $C_{23}H_{24}N_5O_4$: 434.1823; found 434.1829. Rt: 12.19 min.

Figure S8: Structure of ((2R,3R,4S,5R)-2-(6-((4-fluorobenzyl)amino)-9H-purin-9-yl)-5-(hydroxymethyl)tetrahydrofuran-3,4-diol) (**2c**). Overlap of 2D-HMBC spectrum in blue, 2D-HSQC in red/fuchsia and 1D ^{1}H spectrum in green.

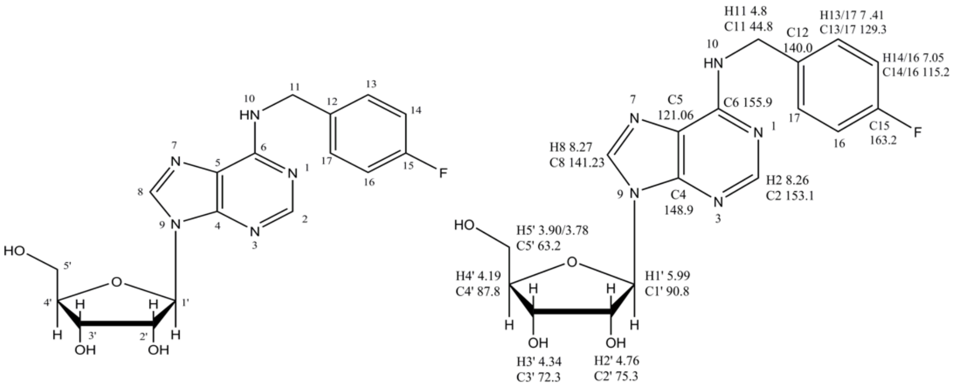

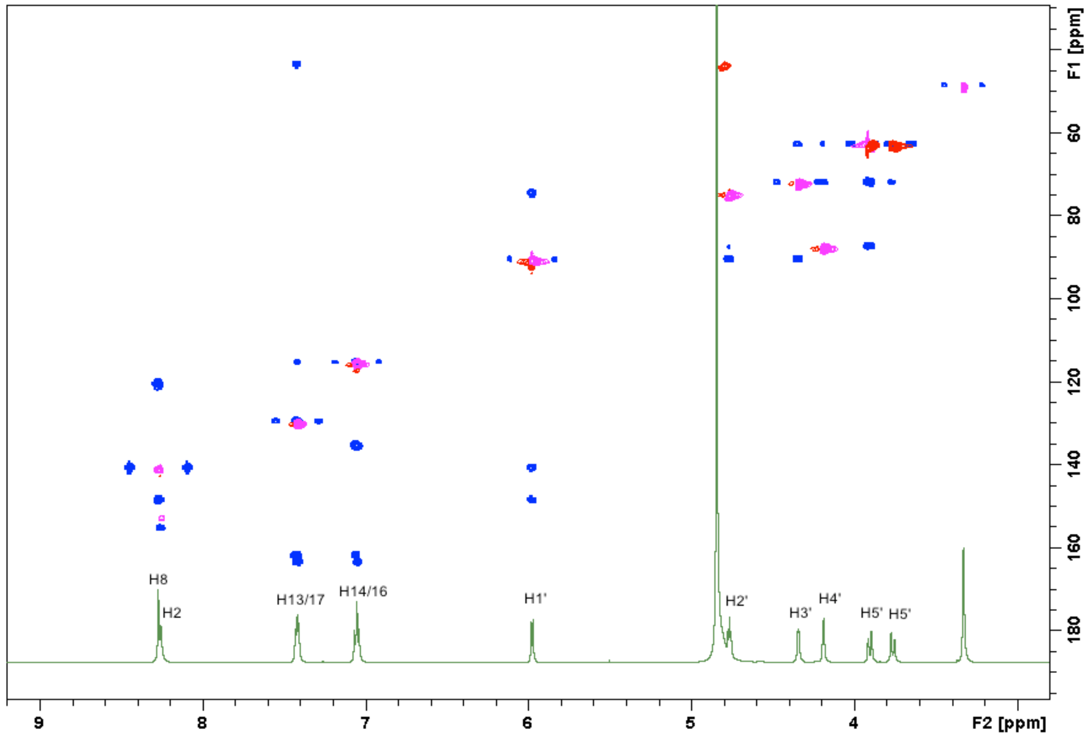

^{1}H NMR (600 MHz, MeOD): δ 8.29 (d, J = 9.8 Hz, 4H), 7.45 (dd, J = 7.9, 5.7 Hz, 4H), 7.08 (t, J = 8.7 Hz, 4H), 6.00 (d, J = 6.4 Hz, 2H), 4.81–4.77 (m, 3H), 4.37 (dd, J = 4.7, 2.3 Hz, 2H), 4.21(d, J = 2.1 Hz, 2H), 3.93 (dd, J = 12.5, 2.0 Hz, 2H), 3.79 (dd, J = 12.5, 2.2 Hz, 2H) ppm. ^{13}C NMR (126 MHz, MeOD): δ 163.2, 155.9, 153.1, 148.9, 141.2, 140.0, 129.3, 121.0, 115.2, 90.8, 87.8, 75.3, 72.3, 63.2, 44.8. HRMS (ESI-Q-TOF) m/z $[M + H]^+$ calcd for $C_{17}H_{19}N_5O_4$: 376.1416; found 376.1411. Rt: 9.00 min.

Figure S9: Structure of ((2R,3R,4S,5R)-2-(6-((4-bromobenzyl)amino)-9H-purin-9-yl)-5-(hydroxymethyl)tetrahydrofuran-3,4-diol) (**2d**). Overlap of 2D-HMBC spectrum in blue, 2D-HSQC in red/fuchsia and 1D ^{1}H spectrum in green.

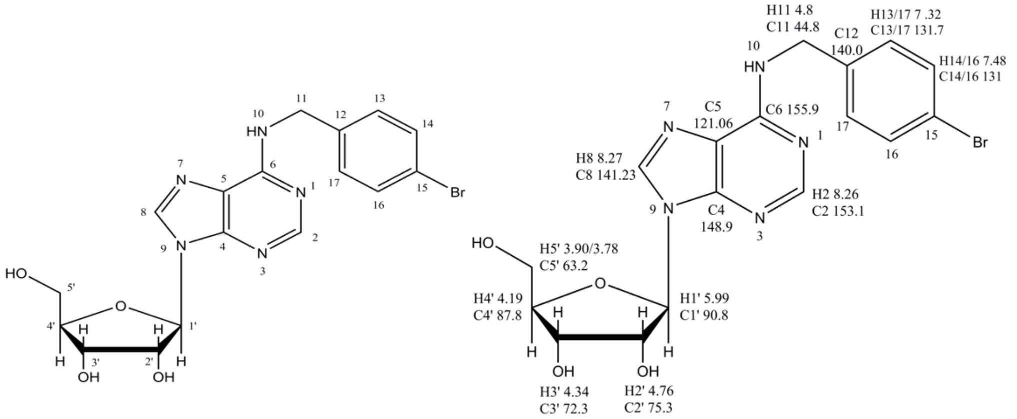

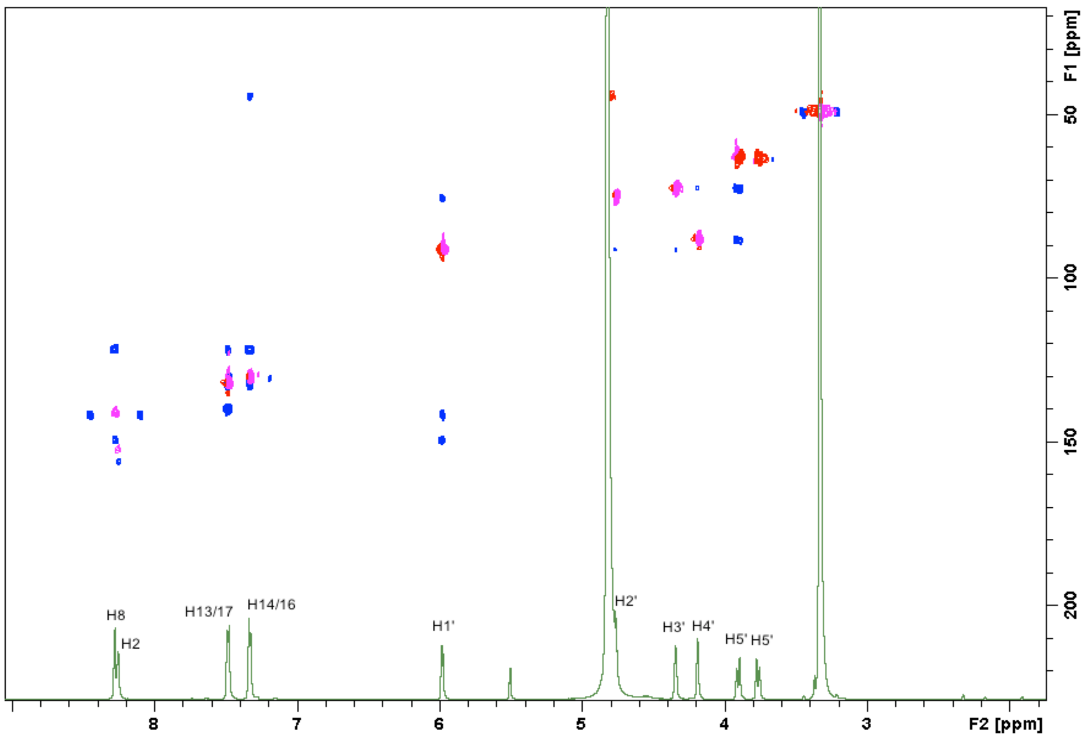

^{1}H NMR (600 MHz, MeOD): δ 8.32 (d, J = 13.8 Hz, 2H), 7.53 (d, J = 7.8 Hz, 2H), 7.38 (d, J = 7.7 Hz, 2H), 6.03 (d, J = 5.9 Hz, 1H), 4.82 (d, J = 5.4 Hz, 2H), 4.39 (s, 1H), 4.24 (s, 1H), 3.96 (d, J = 12.5 Hz, 1H), 3.82 (d, J = 12.5 Hz, 1H) ppm. ^{13}C NMR (126 MHz, MeOD): δ 155.9, 153.1, 148.9, 141.2, 140.0, 131.7, 131.0, 121.0, 90.8, 87.8, 75.3, 72.3, 63.2, 44.8. HRMS (ESI-Q-TOF) m/z $[M + H]^{+}$ calcd for $C_{17}H_{19}BrN_5O_4$: 436.0615; found 436.0611. Rt: 10.53 min.

Figure S10: Structure of ((2R,3S,4R,5R)-2-(hydroxymethyl)-5-(6-((4-methylbenzyl)amino)-9H-purin-9-yl)tetrahydrofuran-3,4-diol) (**2e**). Overlap of 2D-HMBC spectrum in blue, 2D-HSQC in red/fuchsia and 1D ^{1}H spectrum in green.

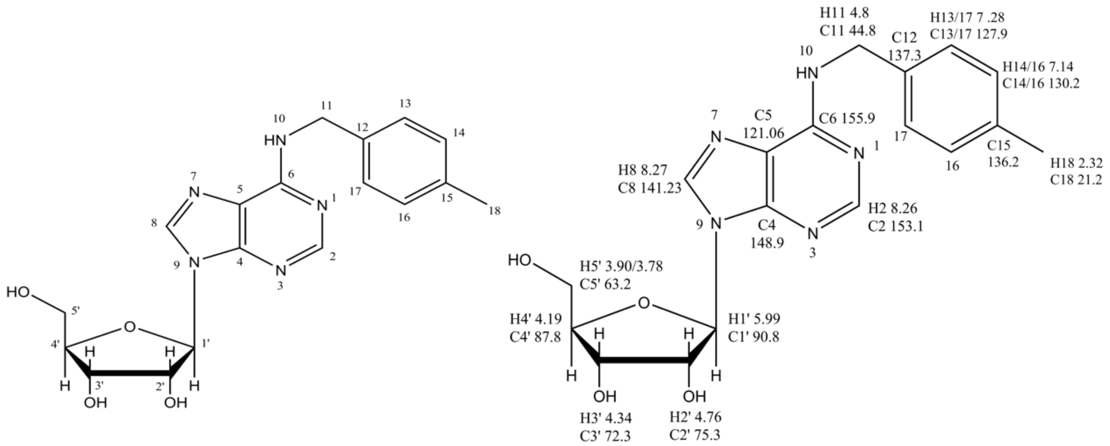

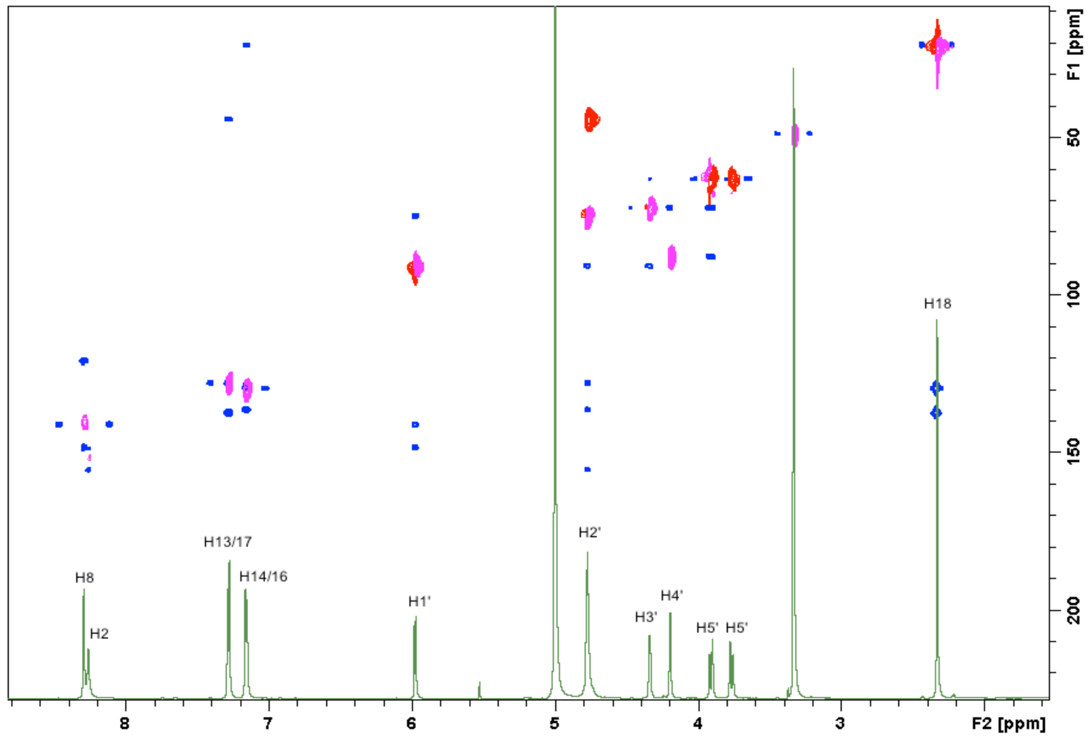

^{1}H NMR (600 MHz, MeOD): δ 8.15 (d, J = 18.7 Hz, 2H), 7.15 (d, J = 7.7 Hz, 2H), 7.03 (d, J = 7.5 Hz, 2H), 5.85 (d, J = 6.5 Hz, 1H), 4.64 (t, J = 5.4 Hz, 3H), 4.21 (d, J = 2.8 Hz, 1H), 4.06 (s, 1H), 3.78 (d, J = 11.6 Hz, 1H), 3.64 (d, J = 11.3 Hz, 1H) ppm. ^{13}C NMR (126 MHz, MeOD): δ 155.9, 153.1, 148.9, 141.2, 136.2, 130.2, 127.9, 121.0, 90.8, 87.8, 75.3, 72.3, 63.2, 44.8, 21.2. HRMS (ESI-Q-TOF) m/z $[M + H]^{+}$ calcd for $C_{18}H_{22}N_5O_4$: 372.1666; found 372.1661. Rt: 9.73 min.

Figure S11: Structure of ((2R,3R,4S,5R)-2-(6-((4-(tert-butyl)benzyl)amino)-9H-purin-9-yl)-5-(hydroxymethyl)tetrahydrofuran-3,4-diol) (**2f**). Overlap of 2D-HMBC spectrum in blue, 2D-HSQC in red/fuchsia and 1D ^{1}H spectrum in green.

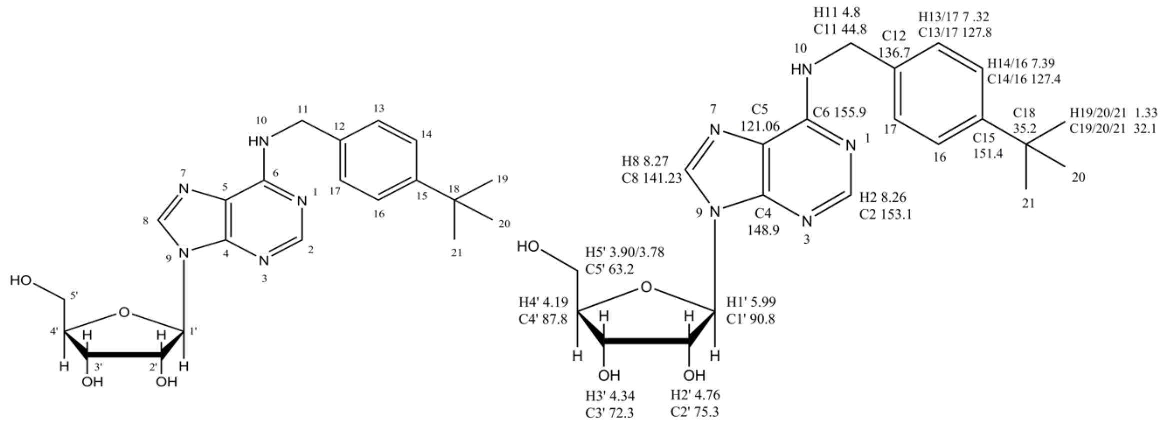

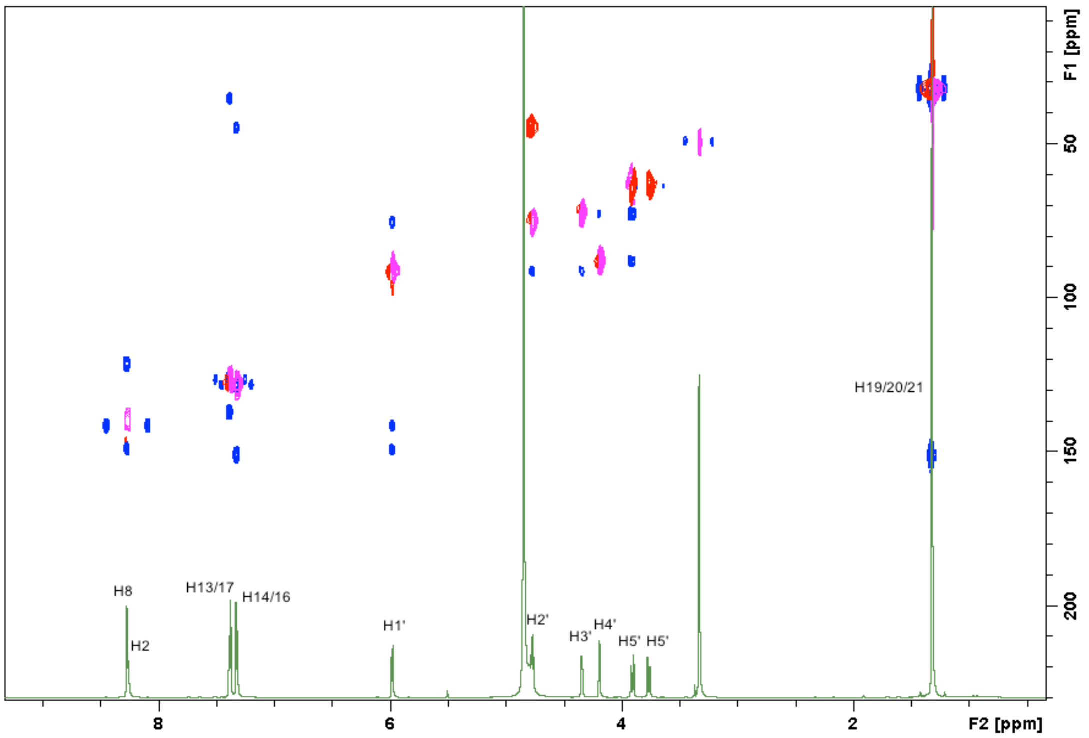

^{1}H NMR (600 MHz, MeOD): δ 8.29 (d, J = 7.6 Hz, 2H), 7.41 (d, J = 7.9 Hz, 2H), 7.35 (d, J = 8.0 Hz, 2H), 6.00 (d, J = 6.3 Hz, 1H), 4.81–4.77 (m, 2H), 4.39–4.34 (m, 1H), 4.21 (s, 1H), 3.93 (d, J = 12.5 Hz, 1H), 3.79 (d, J = 12.5 Hz, 1H) ppm. ^{13}C NMR (126 MHz, MeOD): δ 155.9, 153.2, 151.4, 148.9, 141.2, 136.7, 127.8, 127.4, 121.0, 90.8, 87.8, 75.3, 72.3, 63.2, 44.8, 35.2, 32.1. HRMS (ESI-Q-TOF) m/z $[M+H]^{+}$ calcd for $C_{21}H_{28}N_5O_4$: 414.2136; found 414.2140. Rt: 12.61 min.

Figure S12: Structure of ((2R,3S,4R,5R)-2-(hydroxymethyl)-5-(6-((quinolin-2-ylmethyl)amino)-9H-purin-9-yl)tetrahydrofuran-3,4-diol) (**2g**). Overlap of 2D-HMBC spectrum in blue, 2D-HSQC in red/fuchsia and 1D ^{1}H spectrum in green.

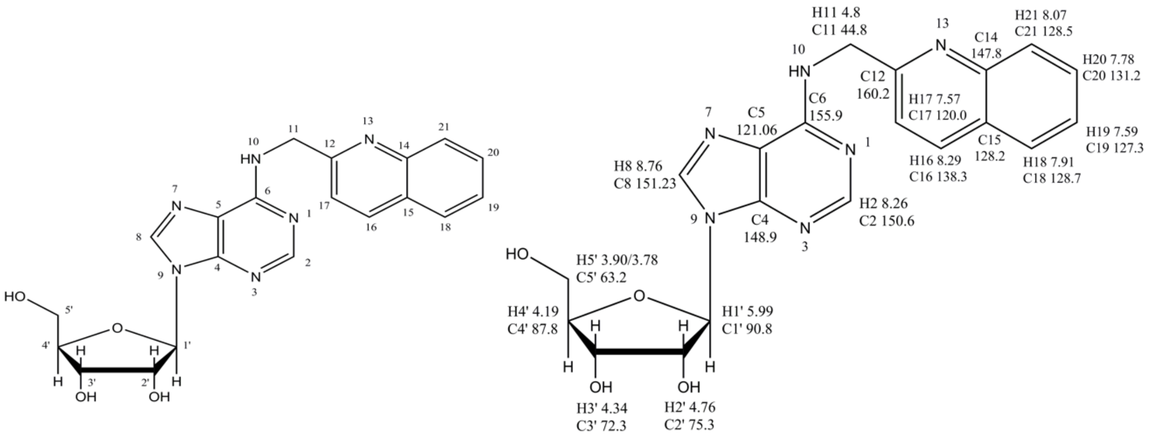

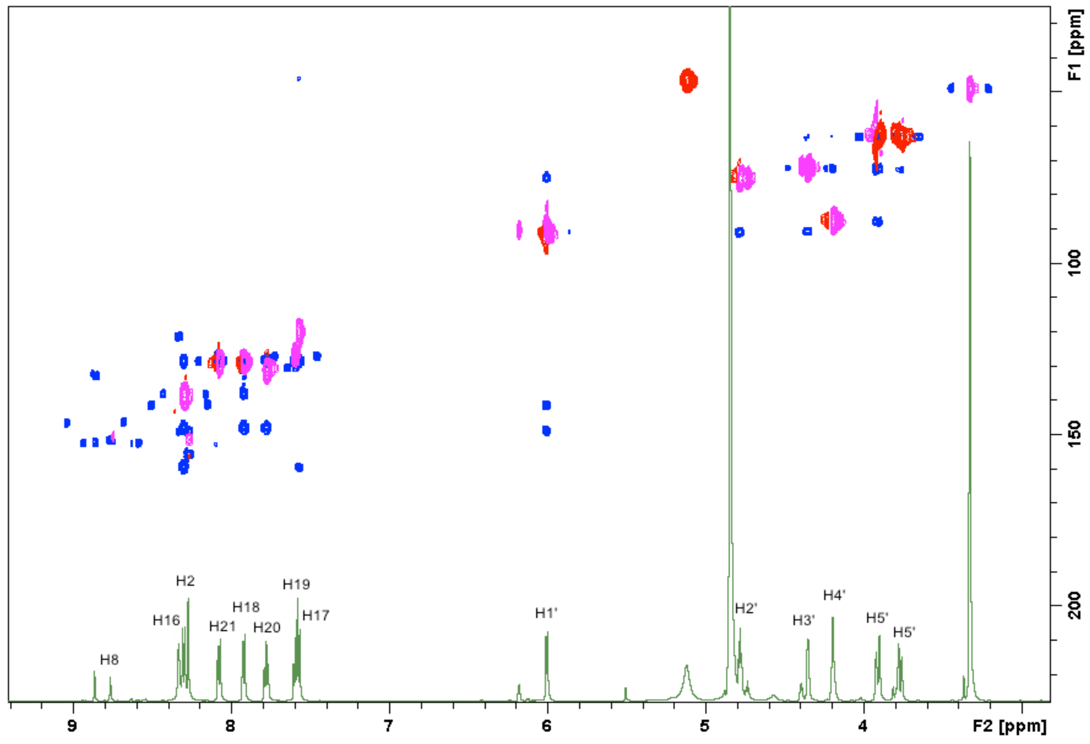

^{1}H NMR (600 MHz, MeOD): δ 8.33 (dd, J = 22.2, 13.7 Hz, 9H), 8.10 (d, J = 8.5 Hz, 3H), 7.95 (d, J = 8.1 Hz, 3H), 7.80 (t, J = 7.6 Hz, 3H), 7.61 (dd, J = 16.0, 8.1 Hz, 6H), 6.03 (d, J = 6.3 Hz, 3H), 4.81 (t, J = 5.6 Hz, 4H), 4.38 (d, J = 2.3 Hz, 3H), 4.22 (s, 4H), 3.93 (d, J = 12.4 Hz, 4H), 3.81 (dd, J = 19.7, 12.5 Hz, 4H) ppm. ^{13}C NMR (126 MHz, MeOD): δ 160.2, 155.9, 150.6, 151.2, 148.9, 147.8, 138.3, 131.2, 128.7, 128.5, 128.2, 127.3, 121.0, 120.0, 90.8, 87.8, 75.3, 72.3, 63.2, 44.8. HRMS (ESI-Q-TOF) m/z [M + H] $^{+}$ calcd for $C_{20}H_{21}N_6O_4$: 409.1619; found 409.1612. Rt: 6.93 min.

Figure S13: Structure of ((2R,3R,4S,5R)-2-(6-((4-chlorobenzyl)amino)-9H-purin-9-yl)-5-(hydroxymethyl)tetrahydrofuran-3,4-diol) (**2h**). Overlap of 2D-HMBC spectrum in blue, 2D-HSQC in red/fuchsia and 1D ^{1}H spectrum in green.

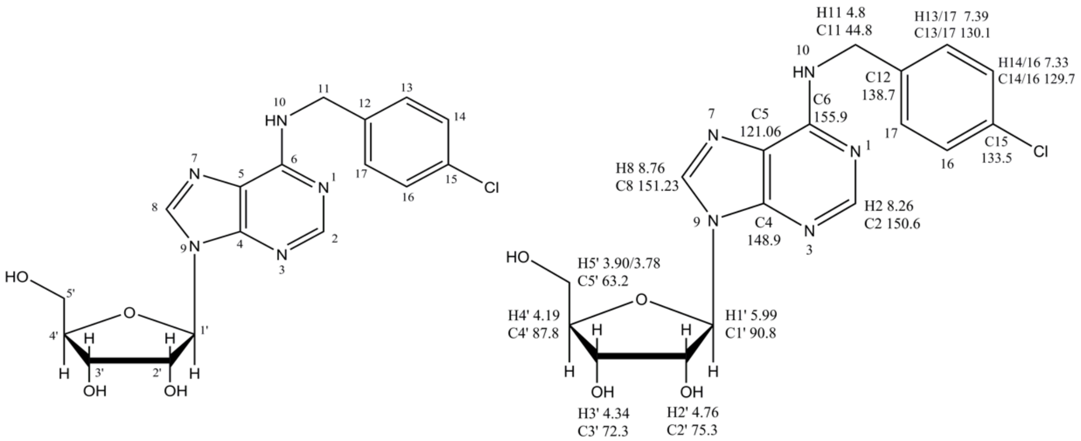

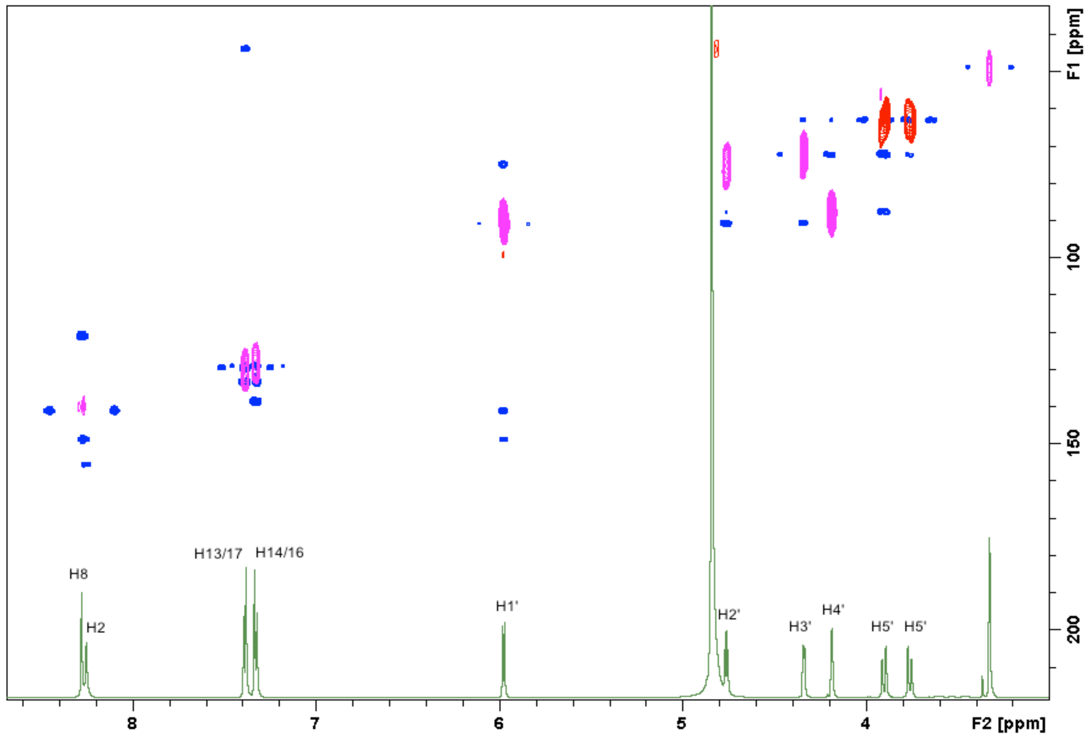

^{1}H NMR (600 MHz, MeOD): δ 8.29 (d, J =15.4 Hz, 2H), 7.42 (d, J = 8.3 Hz, 2H), 7.36 (d, J = 8.4 Hz, 2H), 6.00 (d, J = 6.4 Hz, 1H), 4.81–4.77 (m, 1H), 4.37 (dd, J = 5.0, 2.5 Hz, 1H), 4.21 (d, J = 2.4 Hz, 1H), 3.93 (dd, J = 12.5, 2.3 Hz, 1H), 3.79 (dd, J = 12.5, 2.5 Hz, 1H) ppm. ^{13}C NMR (126 MHz, MeOD): δ 155.9, 151.2, 150.6, 148.9, 138.7, 133.5, 130.1, 129.7, 121.0, 90.8, 87.8, 75.3, 72.3, 63.2, 44.8. HRMS (ESI-Q-TOF) m/z $[M + H]^{+}$ calcd for $C_{17}H_{19}ClN_5O_4$: 392.1120; found 392.1127. Rt:10.34 min.

Figure S14: Structure of ((2R,3S,4R,5R)-2-(hydroxymethyl)-5-(6-((4-nitrobenzyl)amino)-9H-purin-9-yl)tetrahydrofuran-3,4-diol) (**2i**). Overlap of 2D-HMBC spectrum in blue, 2D-HSQC in red/fuchsia and 1D ^{1}H spectrum in green.

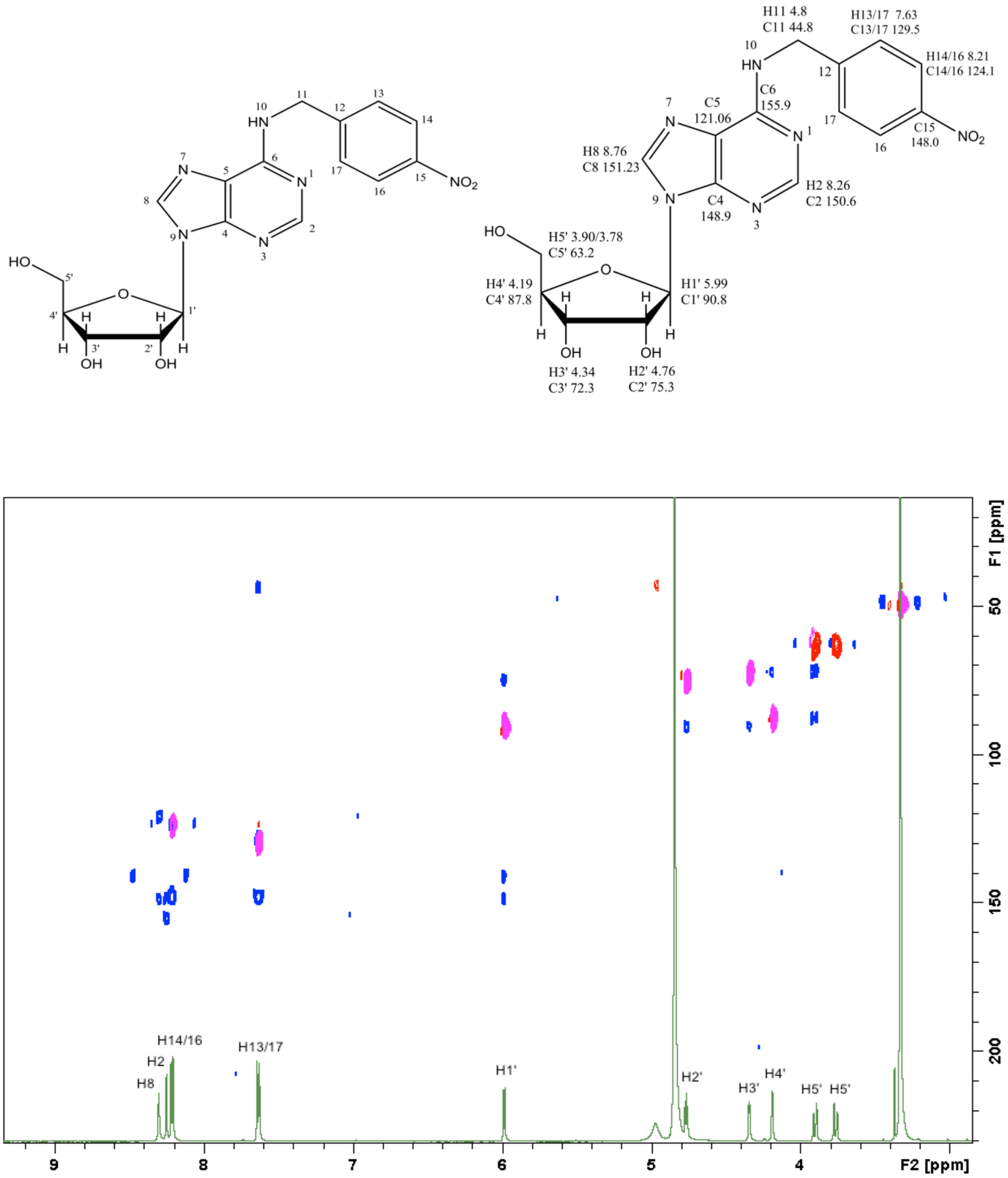

^{1}H NMR (600 MHz, MeOD): δ 8.33 (s, 1H), 8.28 (s, 1H), 8.25 (s, 1H), 8.23 (s, 1H), 7.66 (d, J = 8.6 Hz, 2H), 6.01 (d, J = 6.4 Hz, 1H), 4.81–4.77 (m, 1H), 4.37 (dd, J = 5.0, 2.5 Hz, 1H), 4.22 (d, J = 2.4 Hz, 1H), 3.93 (dd, J = 12.5, 2.4 Hz, 1H), 3.79 (dd, J = 12.5, 2.6 Hz, 1H) ppm. ^{13}C NMR (126 MHz, MeOD): δ 155.9, 151.2, 150.6, 148.9, 148.0, 129.5, 124.1, 121.0, 90.8, 87.8, 75.3, 72.3, 63.2, 44.8. HRMS (ESI-Q-TOF) m/z $[M + H]^{+}$ calcd for $C_{17}H_{19}N_6O_6$: 403.1361; found 403.1354. Rt: 8.84 min.

Figure S15: Structure of ((2R,3S,4R,5R)-2-(hydroxymethyl)-5-(6-((4-methoxybenzyl)amino)-9H-purin-9-yl)tetrahydrofuran-3,4-diol) (**2j**). Overlap of 2D-HMBC spectrum in blue, 2D-HSQC in red/fuchsia and 1D ^{1}H spectrum in green.

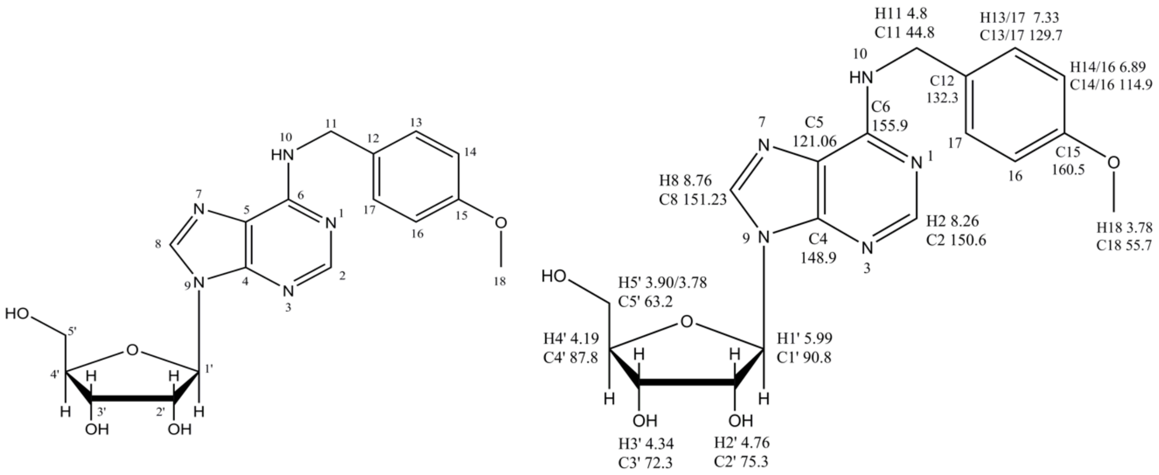

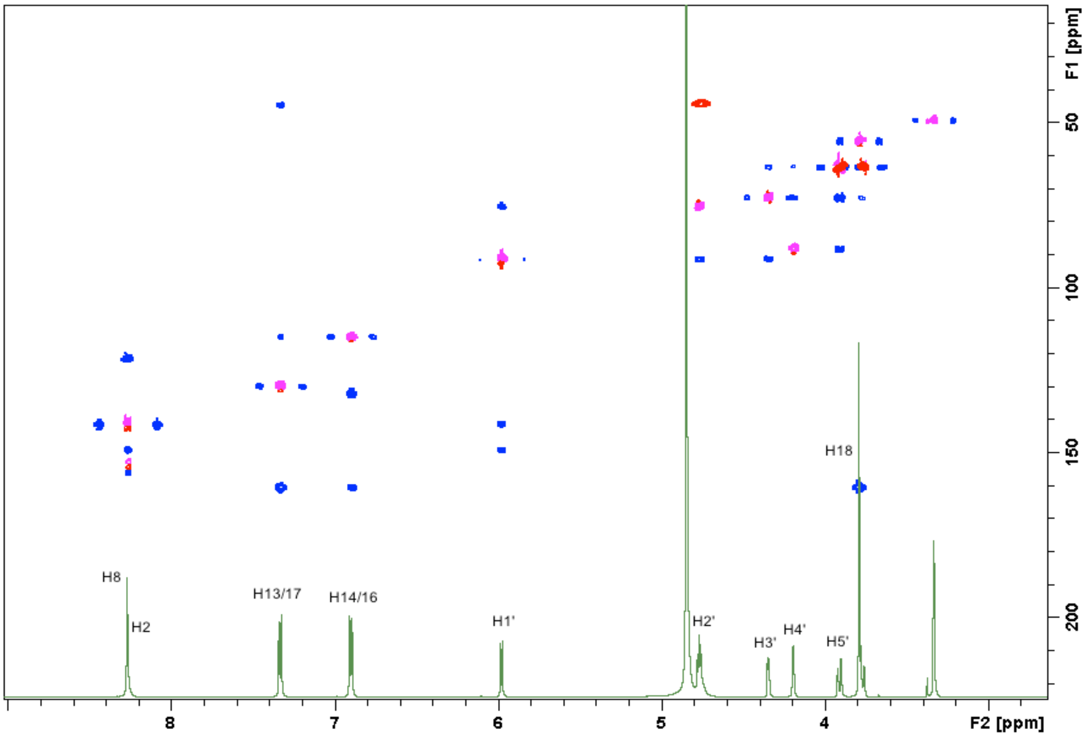

^{1}H NMR (600 MHz, MeOD): δ 8.30 (s, 2H), 7.36 (d, J = 8.5 Hz, 2H), 6.92 (d, J = 8.6 Hz, 2H), 6.00 (d, J = 6.4 Hz, 1H), 4.82–4.77 (m, 2H), 4.37 (dd, J = 5.0, 2.5 Hz, 1H), 4.22 (d, J = 2.4 Hz, 1H), 3.93 (dd, J = 12.5, 2.3 Hz, 1H), 3.82 (s, 3H), 3.79 (dd, J = 12.6, 2.6 Hz, 1H) ppm. 13C NMR (126 MHz, MeOD): δ 160.5, 155.9, 151.2, 150.96, 148.9, 132.3, 129.7, 121.0, 114.9, 90.8, 87.8, 75.3, 72.3, 63.2, 55.7, 44.8. HRMS (ESI-Q-TOF) m/z [M + H] $^+$ calcd for $C_{18}H_{22}N_5O_5$: 388.1615; found 388.1608. Rt: 8.93 min.

Figure S16: Structure of ((2R,3R,4S,5R)-2-(6-((4-ethoxybenzyl)amino)-9H-purin-9-yl)-5-(hydroxymethyl)tetrahydrofuran-3,4-diol) (**2k**). Overlap of 2D-HMBC spectrum in blue, 2D-HSQC in red/fuchsia and 1D ^{1}H spectrum in green.

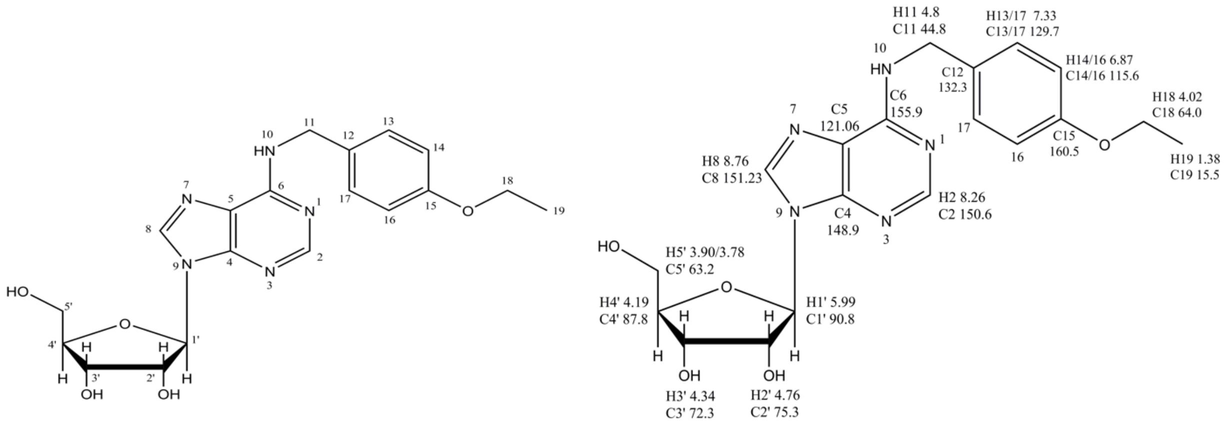

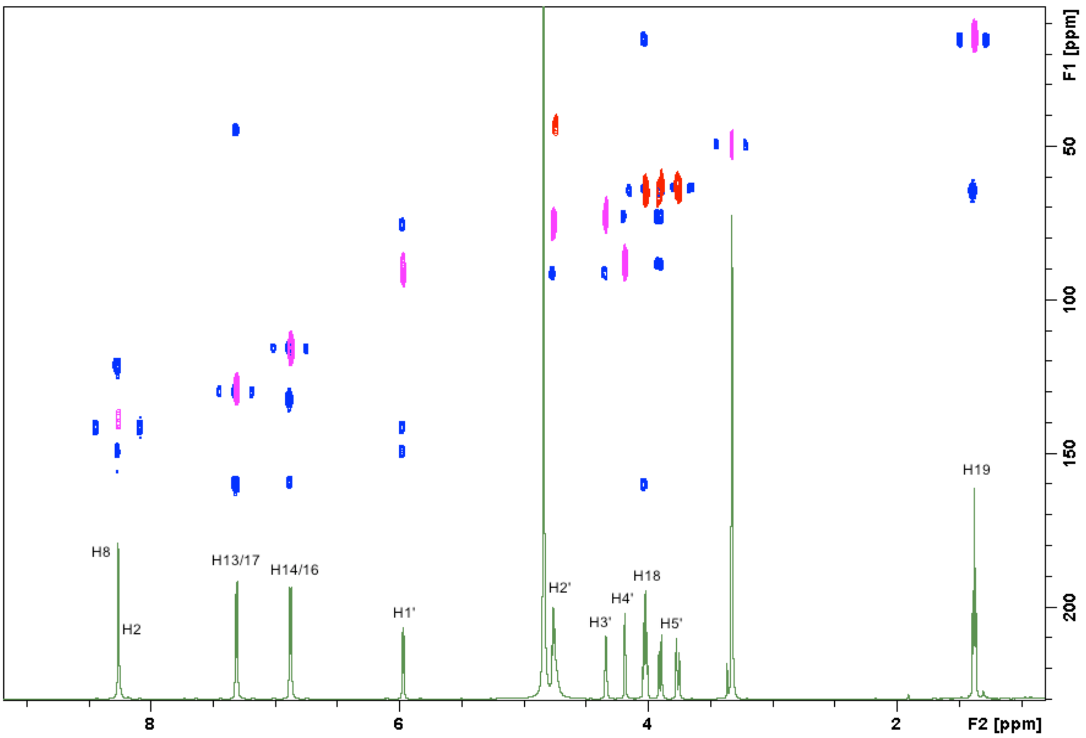

^{1}H NMR (600 MHz, MeOD): δ 8.29 (s, 2H), 7.34 (d, J = 7.9 Hz, 2H), 6.91 (d, J= 7.8 Hz, 2H), 6.00 (d, J = 6.3 Hz, 1H), 4.81–4.77 (m, 2H), 4.38–4.35 (m, 1H), 4.21 (s, 1H),4.05 (q, J = 6.8 Hz, 2H), 3.93 (d, J = 12.5 Hz, 1H), 3.79 (d, J = 12.5 Hz, 1H) ppm. ^{13}C NMR (126 MHz, MeOD): δ 160.5, 155.9, 151.2, 150.6, 148.9, 132.3, 121.0, 129.7, 115.6, 90.8, 87.8, 75.3, 72.3, 64.0, 63.2, 55.7, 44.8, 15.5. HRMS (ESI-Q-TOF) m/z $[M+H]^+$ calcd for $C_{19}H_{24}N_5O_5$: 402.1772; found 402.1779. Rt: 9.66 min.

Figure S17: Structure of ((2R,3R,4S,5R)-2-(6-((3,5-bis(trifluoromethyl)benzyl)amino)-9H-purin-9-yl)-5-(hydroxymethyl)tetrahydrofuran-3,4-diol) (**2l**). Overlap of 2D-HMBC spectrum in blue, 2D-HSQC in red/fuchsia and 1D ^{1}H spectrum in green.

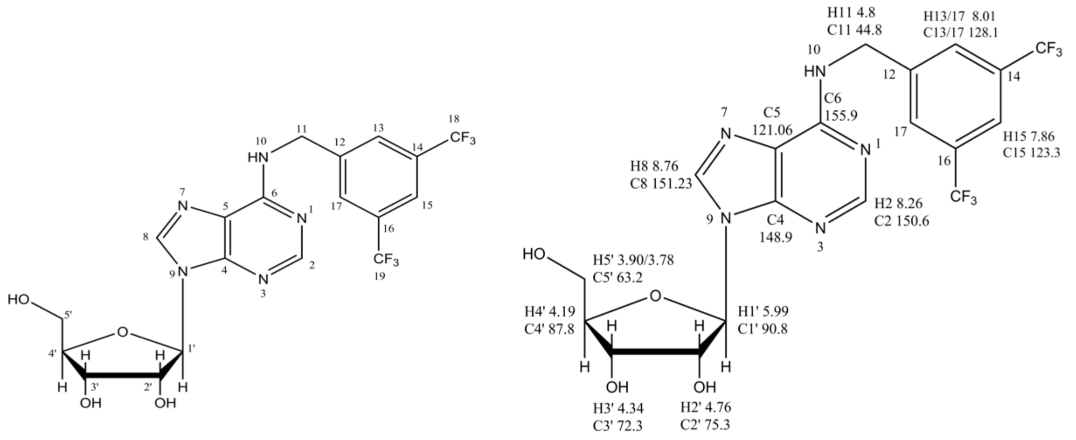

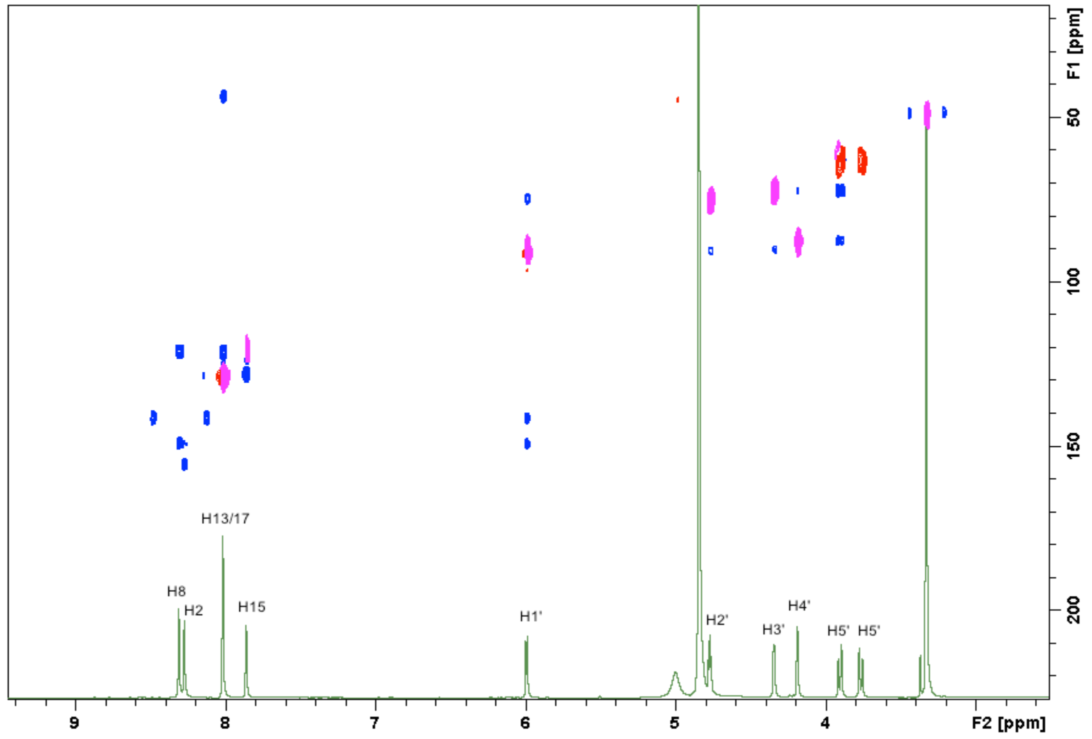

^{1}H NMR (600 MHz, MeOD): δ 8.34 (s, 1H), 8.30 (s, 1H), 8.04 (s, 2H), 7.89 (s, 1H), 6.02 (d, J = 6.3 Hz, 1H), 4.80 (t, J = 5.6 Hz, 1H), 4.38–4.36 (m, 1H), 4.21 (s, 1H), 3.93 (d, J = 12.5 Hz, 1H), 3.79 (d, J = 12.5 Hz, 1H) ppm. ^{13}C NMR (126 MHz, MeOD): δ 155.9, 151.2, 150.6, 148.9, 128.1, 123.3, 121.0, 90.8, 87.8, 75.3, 72.3, 64.0, 63.2, 55.7, 44.8. HRMS (ESI-Q-TOF) m/z [M + H]$^+$ calcd for $C_{19}H_{18}F_6N_5O_4$: 494.1257; found 494.1263. Rt: 13.03 min.

Figure S18: Structure of ((2R,3R,4S,5R)-2-(6-((3,5-dimethoxybenzyl)amino)-9H-purin-9-yl)-5-(hydroxymethyl)tetrahydrofuran-3,4-diol) (**2m**). Overlap of 2D-HMBC spectrum in blue, 2D-HSQC in red/fuchsia and 1D ^{1}H spectrum in green.

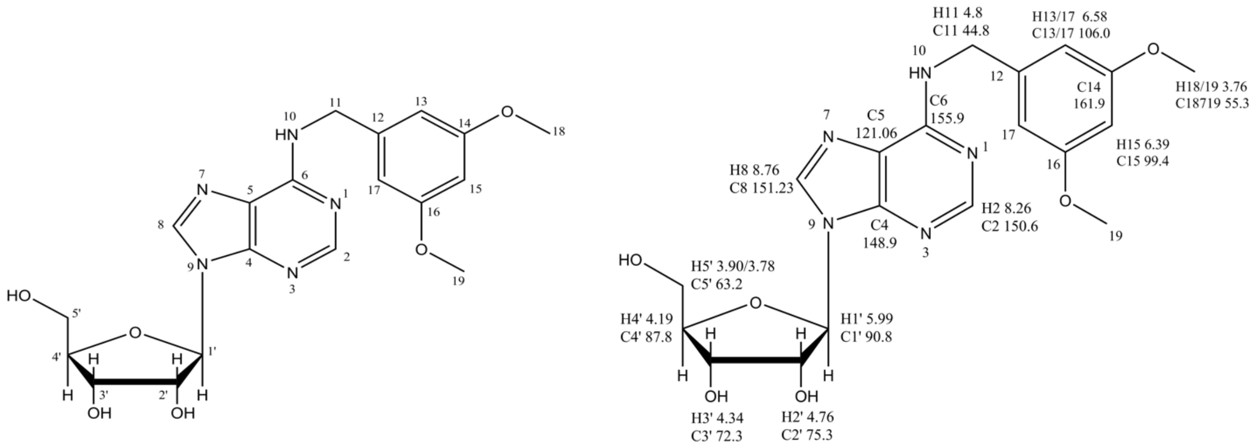

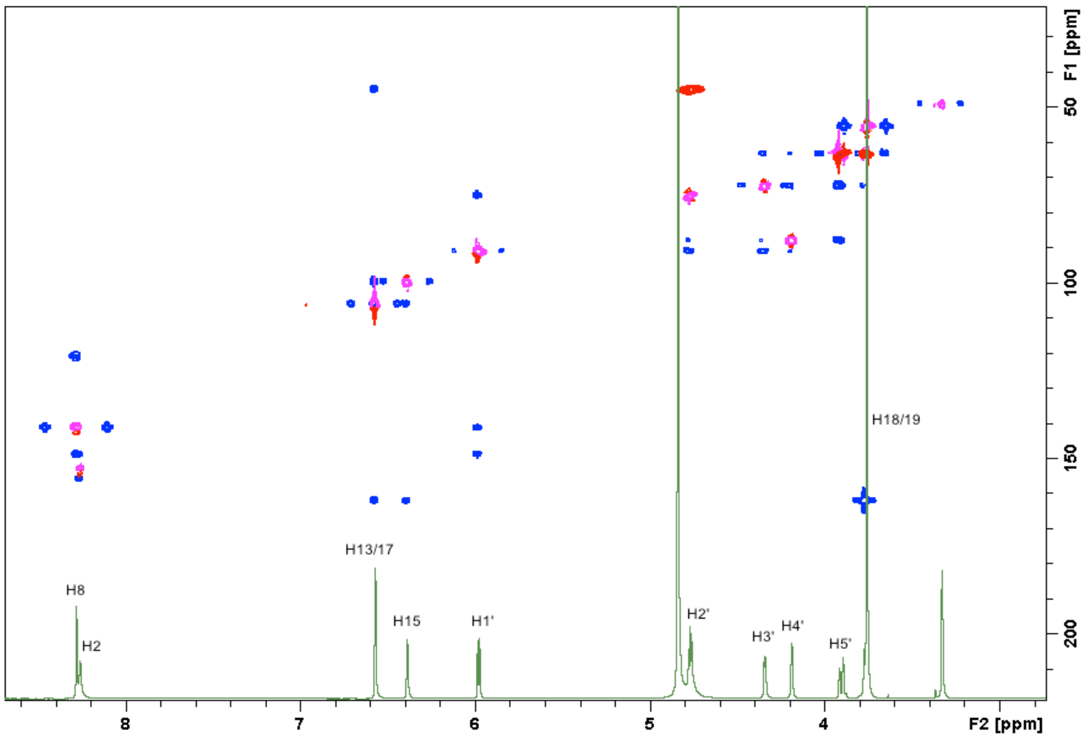

^{1}H NMR (600 MHz, MeOD): δ 8.30 (d, J = 12.4 Hz, 2H), 6.60 (d, J = 1.8 Hz, 2H), 6.42 (s, 1H), 6.01 (d, J = 6.4 Hz, 1H), 4.82–4.78 (m, 2H), 4.37 (dd, J = 4.9, 2.4 Hz, 1H), 4.22 (d, J = 2.3 Hz, 1H), 3.93 (dd, J = 12.5, 2.2 Hz, 1H), 3.80 (d, J = 2.5 Hz, 1H) ppm. ^{13}C NMR (126 MHz, MeOD): δ 161.9, 155.9, 151.2, 150.6, 148.9, 121.0, 106.0, 99.4, 90.8, 87.8, 75.3, 72.3, 64.0, 63.2, 55.7, 55.3, 44.8. HRMS (ESI-Q-TOF) m/z $[M + H]^{+}$ calcd for $C_{19}H_{24}N_5O_6$: 418.1721; found 418.1728. Rt: 9.06 min.
